# Nuclear deformation and dynamics of migrating cells in 3D confinement reveal adaptation of pulling and pushing forces

**DOI:** 10.1126/sciadv.adm9195

**Published:** 2024-08-21

**Authors:** Stefan Stöberl, Johannes Flommersfeld, Maximilian M. Kreft, Martin Benoit, Chase P. Broedersz, Joachim O. Rädler

**Affiliations:** ^1^ Faculty of Physics and Center for NanoScience, Ludwig-Maximilians-University, Geschwister-Scholl-Platz 1, D-80539 Munich, Germany.; ^2^ Arnold Sommerfeld Center for Theoretical Physics and Center for NanoScience, Department of Physics, Ludwig-Maximilians-University Munich, Theresienstraße 37, D-80333 Munich, Germany.; ^3^ Department of Physics and Astronomy, Vrije Universiteit Amsterdam, 1081HV Amsterdam, Netherlands.

## Abstract

Eukaryotic cells show an astounding ability to remodel their shape and cytoskeleton and to migrate through pores and constrictions smaller than their nuclear diameter. However, the relation of nuclear deformation and migration dynamics in confinement remains unclear. Here, we study the mechanics and dynamics of mesenchymal cancer cell nuclei transitioning through three-dimensional compliant hydrogel channels. We find a biphasic dependence of migration speed and transition frequency on channel width, peaking at widths comparable to the nuclear diameter. Using confocal imaging and hydrogel bead displacement, we determine nuclear deformations and corresponding forces during confined migration. The nucleus deforms reversibly with a reduction in volume during confinement. With decreasing channel width, the nuclear shape during transmigration changes biphasically, concomitant with the transitioning dynamics. Our proposed physical model explains the observed nuclear shapes and transitioning dynamics in terms of the cytoskeletal force generation adapting from purely pulling-based to a combined pulling- and pushing-based mechanism with increasing nuclear confinement.

## Teaser

Cells adapt nucleus volume and force generation to effectively migrate through a broad range of spatial confinement.

## INTRODUCTION

Cell migration plays a key role in physiological processes such as wound healing, immune response, and cancer metastasis (*1*–*4*). Cancer cell invasion is an essential step in the metastatic process, owing to the complex interplay of the molecular control of protrusions, reorganization of the cytoskeleton, and interaction with the extracellular matrix (ECM). As cells invade this matrix, they squeeze through tight meshwork forming confinements ranging from less than 1 μm up to tens of micrometers (*5*, *6*). By invading constrictions smaller than the nuclear diameter, the nucleus is required to deform to the size of the constriction (*7*–*9*). The need of physical deformation emphasizes the importance of nuclear flexibility in cellular invasion into the ECM network (*7*, *10*). Migration in confining environments raises the general question, whether and how cells can control their nuclear properties and force generation machinery to overcome such varying spatial constrictions.

Over the past two decades, an increasing number of studies have focused on the role of confinement in cell migration. In general, restriction of two-dimensional (2D) migration to 1D patterned lanes or microstructured channels was found to accelerate migration (*11*, *12*). However, in narrow 3D constrictions where nuclear deformation is required, the translocation of the nucleus was identified as the rate-limiting step during migration in early studies (*13*–*16*). Initially, the role of the nucleus in confined migration was reduced to that of a passive cargo, whereby its viscoelastic properties were important for cellular migration (*7*, *17*, *18*). Recent studies using flat silicon microcantilevers to confine cells, however, have presented evidence that besides passively deforming, cells can use their nucleus to sense confinement and actively change nuclear shape (*19*, *20*). In contrast to the externally imposed confinement in these studies, cells typically “self-impose” their confinement when spontaneously migrating within and through tight pores. It is still unclear whether such active mechanisms play a role during self-imposed confined migration.

To study self-imposed confined cell migration quantitatively, artificial microfluidic assays are commonly used. Notably, microfabricated hydrogel-based migration assays (*18*, *21*–*23*) and trans-well migration assays (*17*, *24*, *25*) have proven valuable to understand the effect of nuclear mechanical properties on cell migration and survival at the single-cell level (*17*). Long, linear polydimethylsiloxane (PDMS) channels (*14*, *26*, *27*) are frequently used to investigate the migration dynamics of cells physically confined within a controlled experimental environment. However, to investigate cell entry into constrictions, short circular pillar-lined PDMS channels have proven instrumental, as they make it possible to study the effect of confinement on migration without perturbing the overall mode of migration (*28*, *29*).

While the importance of the nucleus in confined migration has become clear, it remains debated how the cytoskeleton is involved in deforming and maneuvering the nucleus into constrictions. Several studies identified a dominant contribution of contractile actomyosin fibers in front of the nucleus generating “pulling” forces that are transmitted to the nucleus via the LINC complex anchoring actin via nesprin-2 to the nuclear membrane (*29*–*31*). In contrast, recent work that analyses the cytoskeleton structure of cells inside and outside of physical confinement, however, revealed an accumulation of actin as well as myosin and thus an increase in cortical contractility in the rear of confined cells (*32*, *33*). This was preempted by an increase in Ca^2+^ in the cytosol that was also observed previously in response to nuclear deformations (*19*, *20*). This points toward the possibility of the involvement of an osmotic “pushing” force in confined cell migration, which they further support with laser ablation experiments (*32*). Despite this recent progress, it remains unclear to what extent these pushing forces act onto the nucleus, how the balance between pulling and pushing forces depends on the degree of confinement, and how this interplay determines the overall migration dynamics of cells that self-impose physical confinement by spontaneously entering tight constrictions (*7*, *19*, *20*, *34*).

Here, we take a biophysical approach to address these questions: We introduce arrays of compliant 3D hydrogel microcavities to monitor single cancer cells spontaneously migrating through a constricting channel. For the cancerous cell line (MDA-MB-231) used in this study, we observe a biphasic behavior of cell transition rates as a function of constriction width with rates peaking around a channel width comparable to the nuclear diameter. We analyze the repeated 3D nuclear deformation as well as wall deformation during migration through the compliant 3D channel to quantify the forces involved in nuclear translocation during migration. Our analysis confirms the notion that pushing forces increase in confinement, which coincides with an increase in Ca^2+^, confirming that the proposed biochemical pathway from calcium release to myosin accumulation in the cell rear indeed affects the force balance at the nucleus. We then connect this adaptive force generation mechanisms to the emergent migration dynamics by incorporating it in a biophysical model for confined cell migration, which explains the observed dependence of both the cellular migration dynamics and nuclear morphology on channel width. Our findings support the notion that cells actively adapt their cytoskeleton force generation during self-imposed cell migration to generate pushing forces in the cell rear in addition to pulling forces in the cell front to overcome spatial constrictions.

## RESULTS

### A versatile hydrogel-based assay is used to study 3D confined migration

**Fig. 1.**
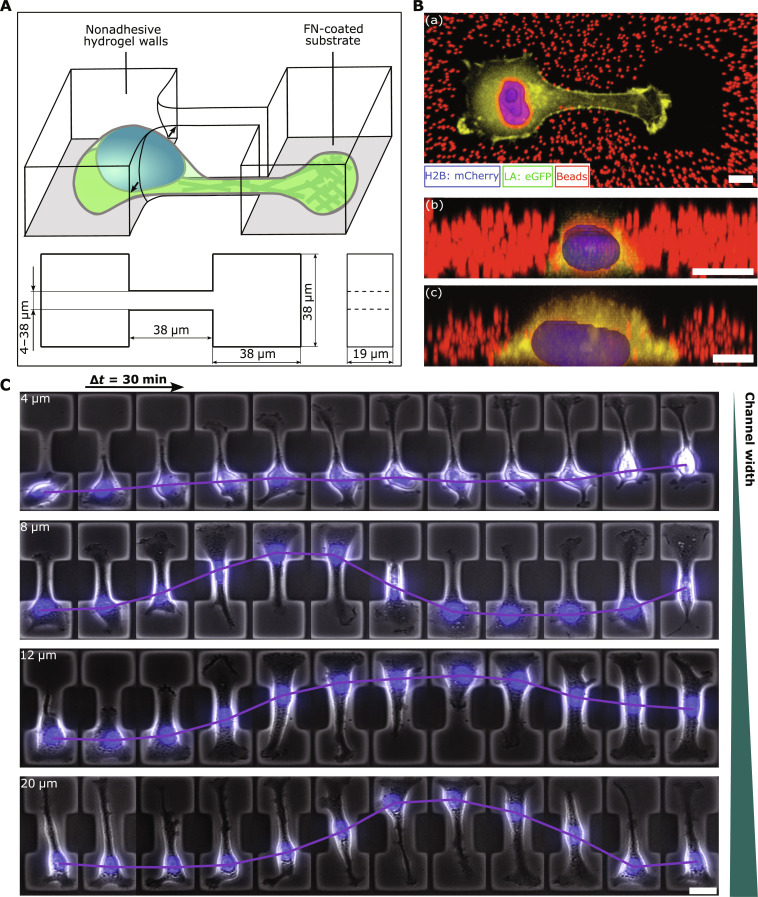
Synthetic hydrogel-based assay to study 3D confined cell migration. (**A**) Schematic depicting of the experimental assay consisting of two fibronectin-coated square chambers (35 μm by 35 μm) connected by a channel. The dumbbell-shaped geometry is surrounded by nonadhesive and soft PEG-NB hydrogel, which gets deformed by cells traversing the constriction. (**B**) Exemplary confocal images show an MDA-MB-231 cell coexpressing fluorescently labeled histones (mCherry-H2B) and F-actin (LifeAct-TagGFP2) confined to the micropattern (a). Embedded fluorescent nanobeads indicate the surrounding PEG-NB hydrogel, which physically confines cells within the cavity (b). As the hydrogel is nonadhesive, cell adhesion is solely restricted to the substrate (c) (scale bar, 10 μm). (**C**) Exemplary time series of MDA-MB-231 cells for varying constriction width (*D* = 3 to 35 μm) (scale bar, 20 μm).

To quantitatively study the influence of physical confinement and nuclear deformations on the migration dynamics of cells, we develop a hydrogel-based migration assay to perform high-throughput cell migration experiments within a controllable geometry (Fig. 1A). Our setup uses 3D dumbbell-shaped microcavities embedded within a 20-μm-thick poly(ethylene glycol)-norbornene (PEG-NB) hydrogel layer. Fluorescent nanobeads mixed into the hydrogel serve as markers to determine forces exerted by cells from the elastic deformation field of the walls (Fig. 1B). The bottom of the cavities is coated with fibronectin to promote cell adhesion, while the PEG-NB hydrogel is nonadhesive to cells (fig. S1). Throughout the experiments, as depicted in Fig. 1Bb, cells are maintained entirely within the cavity walls. In addition, this design ensures that cell adhesion solely occurs at the bottom of the cavity (Fig. 1Bc). Accordingly, the assay is a 3D and deformable generalization of the flat microcontactprinted dumbbell pattern used in prior work (*11*). We use time-lapse phase-contrast microscopy to investigate repeated migration of cells from one cavity site to the other through the deformable channel with defined width. Inside the 3D dumbbells, metastatic human breast cancer cells (MDA-MB-231) migrate spontaneously in mesenchymal mode inside the microcavities (Fig. 1C). At the entrance of the constricting channel, lamellipodia-like protrusions develop repeatedly, occasionally evolving into larger sustained protrusions. While some protrusions retract rapidly, others reach the adjacent unoccupied cavity. In the new cavity, the protrusion widens with an almost half-circular shape, which ultimately results in the transition of the cell body. After the complete cellular transition, the shape and actin distribution of the cytoskeleton randomize (movie S1), leading to a state identical to that preceding the transition. Subsequently, another transition soon follows in the reverse direction, illustrating the reversible nature of this confined migration (movies S2 and S3).

### Cells show biphasic migration rates depending on confinement

We investigate cell migration as a function of confinement widths by fabricating 3D dumbbell arrays with channel width varying over a broad range from 4 to 35 μm. The average nuclear width outside of the channel is 11.62 ± 0.01 μm, where we averaged over different geometries and cells in different stages of their cell cycle. For channel widths wider than this threshold (Fig. 1C, lowest row), cells can typically enter the channel without requiring nuclear deformations. In this case, the migration within the channel shows little persistence, and some cells even change their direction of motion in the middle of the channel without reaching the opposite side of the pattern (movie S2). In channels with intermediate widths smaller than the nuclear width (Fig. 1C, second and third rows), cells exhibit large nuclear deformation (movie S3). After nuclear shape adaption, almost all cells consistently migrate to the other chamber (fig. S18B). For the narrowest channels (Fig. 1C, upper row), unsuccessful attempts of nuclear translocations are frequently observed. In channels where the nucleus becomes confined (<12 μm), we observe an increased fraction of “trapped” cells that typically still form protrusions that extend to the other side of the pattern but are unable to move their nucleus into the channel (Figs. 1C, upper row, and 2A, columns 1 and 2). Our results do not indicate a specific threshold width below which migration is completely inhibited, but rather a gradual reduction in the fraction of migratory cells with diminishing channel width. Moreover, when transitions require large nuclear deformations, the migration behavior markedly differs from dynamics found on flat micropatterned 2D dumbbells, where we previously observed frequent transitions, even for the narrowest bridge widths (*35*). This observation highlights that 3D confinement qualitatively alters cell translocation.

**Fig. 2.**
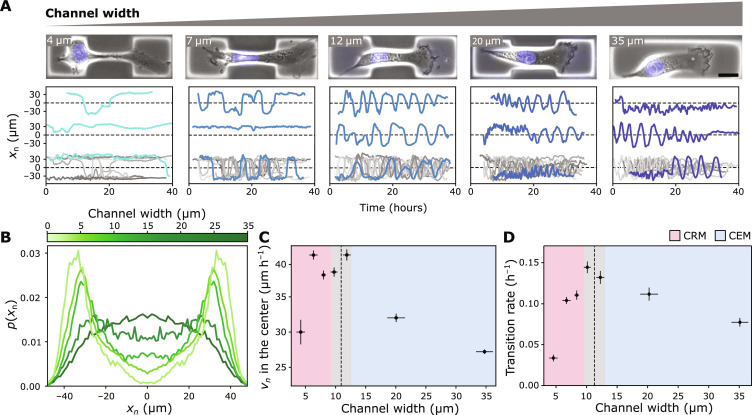
Migration statistics for varying channel widths. (**A**) Representative examples of cell trajectories for different channel widths (from left to right: 4, 7, 12, 20, and 35 μm) (scale bar, 20 μm). (**B**) The distributions of the nuclear position *x*_n_ at different channel widths. (**C**) Average velocity in the center of the pattern (|*x*_n_| ≤ 6 μm). The maximal velocities are observed at an intermediate channel width comparable to the nuclear width. (**D**) Transition rates for varying channel widths. Similar to the nuclear velocities, the highest transition rates are observed at intermediate channel widths. [(C) and (D)] The red region indicates the regime of confinement-reduced migration (CRM) and the blue region indicates confinement-enhanced migration (CEM). The dotted line indicates the average nuclear width, and the gray region is 1 SD. Error bars associated with the *x* axis represent the SD, while error bars associated with the *y* axis represent the SE.

To further investigate this, we evaluate the probability distribution of nuclear positions in our microcavities, the average velocity at the channel center, and the transition rates across varying channel widths. In the absence of confinement, the distribution of nuclear positions along the long axis of the pattern is nearly uniform over large parts of the microcavity (Fig. 2B). With decreasing channel width, the distribution transforms into a double-peaked distribution, with marked maxima in the chambers and a pronounced local minimum located in the center of the confining channel. However, even for channel widths of 4 μm, substantially smaller than the average nuclear diameter, we still observe cells that can successfully move their nucleus through these remarkably tight constrictions. We observe a nonmonotonic dependence of the average velocity on the channel width, with a maximum at a confinement width comparable to the undeformed diameter of the nucleus (Fig. 2C). The overall transition kinetics for cells to enter and transmigrate the constricting channel, quantified by the transition rate, shows a similar biphasic dependence on channel width (Fig. 2D): When starting from the unconfined case and gradually decreasing channel width, we initially observe an increase of the transition rate, followed by a steep drop in the transition rate at channel widths below the nuclear size. We term these the “confinement-enhanced migration” (CEM) and the “confinement-reduced migration” (CRM) regimes. These results illustrate the effect that confinement has on the migration behavior of cells, where it either appears to enhance the rate of transitions at wider channel widths or strongly impede transitions when cells need to physically deform their nuclei in tight constrictions, as observed in other studies (*15*, *36*).

### Nucleus deformation affects migration dynamics

To obtain a better understanding of the underlying dynamics driving the transition from the CEM regime to the CRM regime, we exploit the statistics of nuclear velocities and transition rates obtained from our high-throughput experimental assay. We use the recorded nuclear trajectories to infer a stochastic dynamical model for this confined migration process, enabling us to disentangle deterministic and stochastic components of the dynamics. Both free and confined 2D migration can be described by an underdamped Langevin equation of the form (*11*, *37*–*39*).

$$\frac{dv_{n}}{\mathrm{dt}}=F_{w}\left(x_{n},v_{n}\right)+\sigma_{w}\xi \left(t\right)$$ (1)

where *F_w_*(*x*_n_, *v*_n_) captures how the deterministic acceleration of the nucleus depends on position and velocity in a confinement of width *w*, and the Gaussian white noise ξ(*t*) of strength σ*_w_* accounts for the stochastic nature of cell migration. Here, we extend this approach to 3D confined migration by applying the underdamped Langevin inference algorithm (*38*) on our data (section S3 and figs. S13 to S16).

**Fig. 3.**
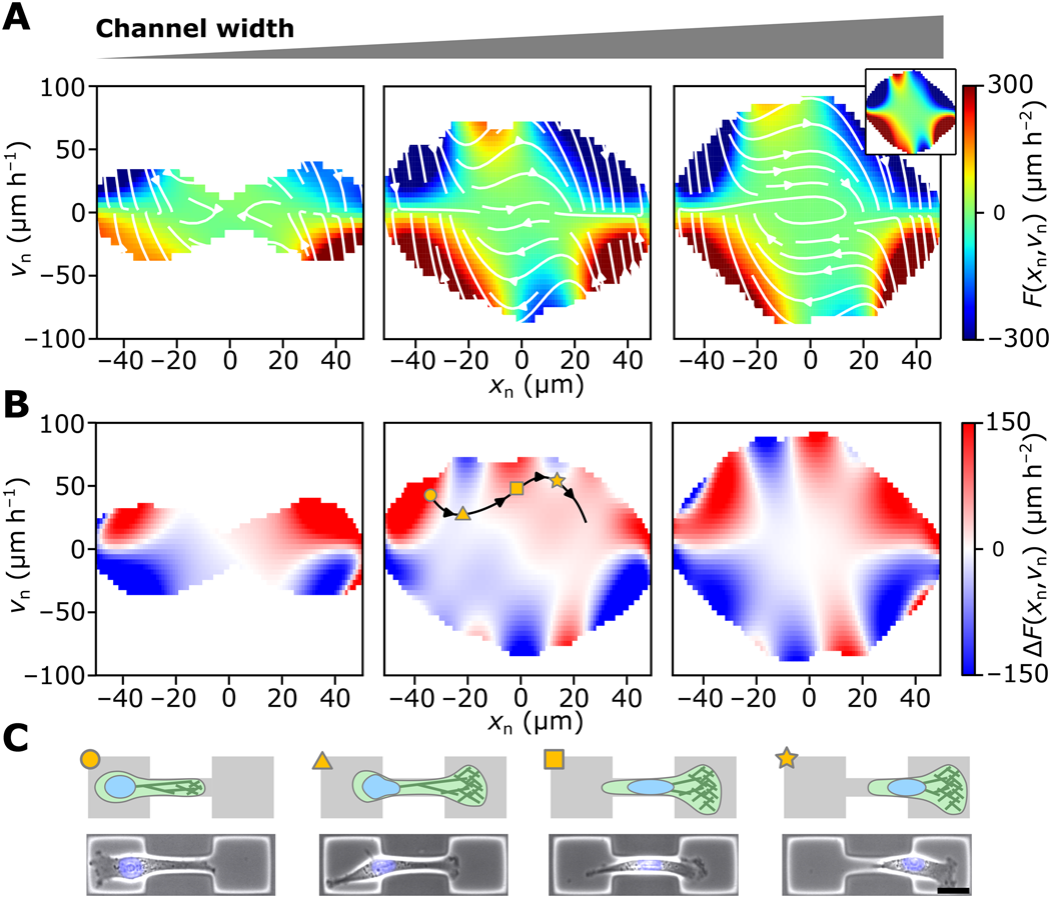
Inferred nonlinear dynamics of the nucleus for varying channel widths (from left to right: 4, 7, and 9 μm). (**A**) The inferred deterministic term *F_w_*(*x*_n_, *v*_n_) within the experimentally sampled region (see Supplementary Materials for details). The outer white region is not sampled in the experiments. Right inset: *F_w_*(*x*_n_, *v*_n_) for a channel width of 12 μm. (**B**) The difference between the deterministic term *F_w_*(*x*_n_, *v*_n_) and the reference term at 12 μm (“Nuclear Confinement Maps”). The black line indicates an exemplary trajectory of a transitioning cell. (**C**) Snapshots of the typical cellular morphology at different points during the transition as indicated in (B) (scale bar, 20 μm).

To understand the impact of the 3D confinement on dynamics, we compare the deterministic terms *F_w_*(*x*_n_, *v*_n_) inferred for different channel widths *w* (Fig. 3A). We focus on the transition from the CEM regime into the CRM regime, such that we only consider the dynamics at channel widths of 12 μm and below. For *w* ≥ 7 μm, we find qualitatively similar models: The nucleus on average strongly decelerates in the center of the two chambers. By contrast, as the nucleus approaches the constricting channel, it accelerates into the channel and ultimately transitions to the other side of the pattern. The structure of these nonlinear dynamics is, at first glance, reminiscent of cells migrating on corresponding 2D micropatterns (*11*). At the narrowest channel width (4 μm), this acceleration region vanishes, consistent with the more stationary behavior that we observed experimentally at this channel width (Fig. 2A).

To better visualize the effect of nuclear deformations on the nonlinear dynamics, we compute the difference Δ*F_w_* = *F*_12μm_ − *F_w_* (Fig. 3B) to compare to the channel width *w* = 12 μm below which nuclear deformations become notable, which we term “Nuclear Confinement Maps” (NCM). At all three considered channel widths, the NCMs show a number of shared qualitative features: A cell that migrates along a typical trajectory from the left to the right chamber (black line in Fig. 3B, center) starts in a region where Δ*F_w_* > 0 (circle in Fig. 3, B and C). In previous work, we found that, on 2D micropatterns, increasing confinement of the protrusion stimulates increasing protrusion growth and thus stronger accelerations of the nucleus toward the channel (*35*). This pronounced region of Δ*F_w_* > 0 could be a signature of this “geometry adaptation” mechanism also being present in 3D confinement. Once the nucleus approaches the channel, it crosses over into a region of Δ*F_w_* < 0 in the NCM (triangle in Fig. 3, B and C). Because the region of Δ*F_w_* < 0 coincides with the point at which further migration requires large nuclear deformations, this suggest that this feature of the NCM may be due to an effective deformation energy barrier that impedes entry into the channel. Once the nucleus is moved into the channel, we again observe a region of acceleration (square in Fig. 3, B and C) that is consistent with an elastic release of the tension that was built up to deform the nucleus in the previous step. If cells reach high enough velocities, they cross through another region of Δ*F_w_* < 0 (star in Fig. 3, B and C) as it leaves the channel. Overall, the NCMs indicate that there is a clear qualitative signature of 3D nucleus confinement that suggest that elastic deformations of the nucleus affect the transmigration dynamics.

### Nuclei reversibly reduce volume during transmigration

To further investigate the role of nuclear mechanics indicated by the NCMs, we analyze the nuclear deformations induced by the confining channel. We find that during transmigration the nuclear shape is strongly deformed depending on the channel width. The nucleus rapidly recovers its original round shape, with a relaxation time of 20 min after exiting from the channel (fig. S10). This observation suggests that, on the relevant timescales in our system (hours), the nuclear response can be considered predominantly elastic, consistent with the dynamics revealed by the NCMs.

**Fig. 4.**
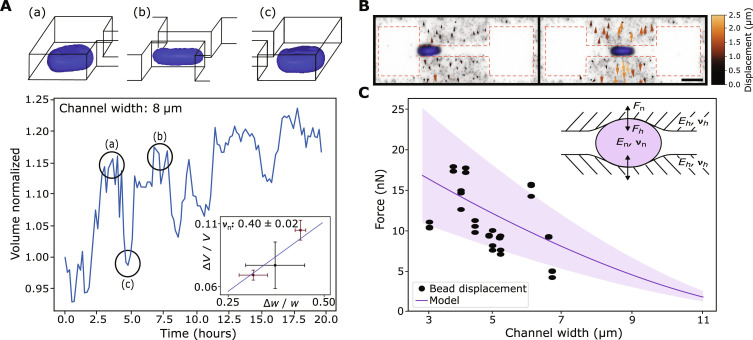
Mechanical properties of cell nuclei in confinement. (**A**) Quantitative nuclear volume analysis of exemplary confocal z-stacks over 20 hours. Nuclear volume decreases in confinement (ii) compared to an unconfined state (i and iii). Data were obtained using the software “arivis Vision4D”. Inset plot: Relative volume change of the nucleus when confined by the channel with three different widths (mean ± SD, *n*_5μm_ = 3; *n*_8μm_ = 4; *n*_10μm_ = 4 from three biological replicates for each channel width) and fit of an elastic model to obtain the Poisson ratio ν of the nucleus (blue line). (**B**) Representative image of the fluorescently labeled nucleus of an MDA-MB-231 cell passing through the soft PEG-NB hydrogel channel. Color-coded arrows indicate the displacement field of fluorescent beads embedded in the hydrogel (scale bar, 20 μm). (**C**) Contact forces between the nucleus to the hydrogel walls as a function of channel width. For comparison, the expected forces are shown on the basis of a Hertz model using independently measured Young’s moduli of the hydrogel and cell nucleus (shaded area indicates 1 SD). Inlet shows schematics of the underlying Hertz model.

To quantify the full 3D nuclear deformation during transition events, we use confocal microscopy (Fig. 4A, fig. S2, and movie S4). Using rendering software, we determine the nuclear volume and ellipsoidal main axis (see Materials and Methods). On longer timescales, over the duration of the experiments, we observe a continuous increase of the nuclear volume of both relaxed and compressed states. This trend corresponds to nuclear growth at a rate 1.1 ± 0.2 $\frac{\%}{h}$ (fig. S2A). As a control, we measure the nuclear volume growth in cavities without any constriction (fig. S2B), which yields a similar nuclear growth rate of 1.0 ± 0.5 $\frac{\%}{h}$ . On shorter timescales, upon the nucleus entering the channel, we observe a transient decrease in nuclear volume of up to 11%, depending on channel width. This phenomenon repeats during subsequent transitions (Fig. 4A and fig. S2A). The volume change of a material under compression can be characterized in terms of the Poisson ratio ν_n_, with ν_n_ = 0.5 characterizing an incompressible material and values below 0.5 indicating that the material reduces volume under compression. From the full 3D deformation under confinement, we determine a Poisson ratio for MDA-MB-231 cell nuclei of ν_n_ = 0.40 ± 0.02 independent of the channel width (Fig. 4A, inset, and section S1.6). Together, these results show that, in the process of translocation, the nucleus behaves as a compressible elastic material indicated by a Poisson ratio distinct from 0.5.

### Nuclear elastic properties are independent of confinement

To determine whether the nuclear elastic properties are affected under strong confinement, we measure the forces exerted onto the channel walls by transitioning cells. We analyze the indentation of the soft hydrogel walls during transitions by tracking the displacement of fluorescent beads that are embedded in the hydrogel (Fig. 4B and movie S5). The observed bead displacements are decomposed into contributions perpendicular and parallel to the migration direction (fig. S4). The perpendicular and tangential forces are estimated by using the maximum bead displacement in the PEG-NB hydrogel (with independently determined Young’s modulus of 2.9 kPa) (fig. S3). Within our detection limit, we do not observe tangential components, which agrees with the nonadhesive property of the PEGylated hydrogel. From the perpendicular component, we find a nonlinear increase of the contact forces exerted by the nucleus with increasing confinement (Fig. 4C). For comparison, we include a theoretical prediction for the contact forces based on an adapted Hertz model (fig. S7). In this scenario, a deformable nucleus, represented by an elastic sphere with a Poisson’s ratio ν_n_ = 0.40 and a Young’s modulus *E*_n_ = 0.4 kPa, indents a soft wall [the Young’s modulus was independently measured by atomic force microscopy (AFM) in the absence of confinement] (fig. S3). The agreement between the estimated contact forces exerted on the nucleus and the predictions of the adapted Hertz model indicate that the elasticity properties of the nucleus do not show noticeable changes during the transmigration of a cell through a 3D constriction.

### Nuclear deformation reveals force adaptation

**Fig. 5.**
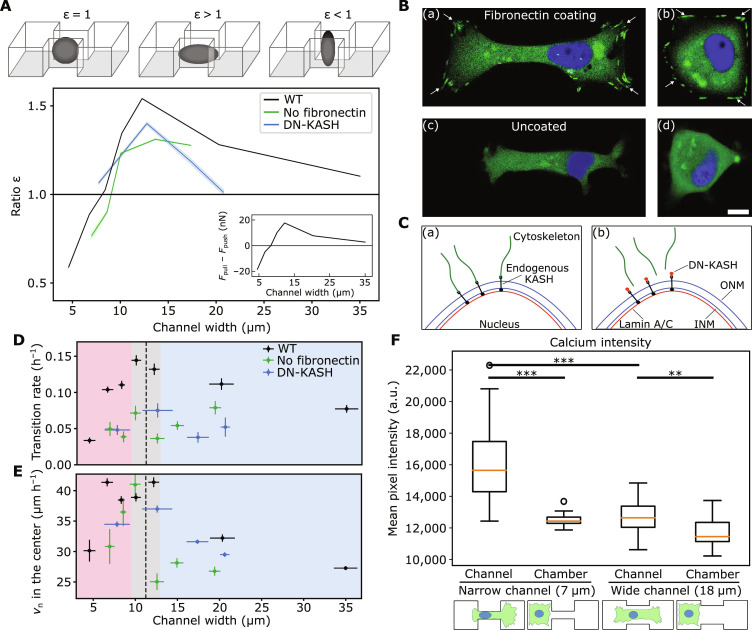
Adaptive cellular force generation in confinement. (**A**) Relative change in the nuclear AR indicates an elongated (ε > 1) or compressed (ε < 1) nuclear shape in the direction of migration, which is consistent with pulling or pushing dominated translocation, respectively. Selectively interfering with cellular pulling forces by removing the fibronectin coating or treatment with DN-KASH results in reduced values of ε at wider channel widths. Inset: Difference between pulling and pushing forces acting on the nucleus. The shaded areas indicate the SEM. WT, wild type. (**B**) Representative images of paxillin-GFP transfected MDA-MB-231 adhered on substrates with [(a) and (b)] and without [(c) and (d)] fibronectin coating. In the presence of fibronectin, paxillin is incorporated in focal adhesion complexes (white arrows) (scale bar, 10 μm). (**C**) Illustration of the linkage between cytoskeleton and nucleus disrupted by the transfection of cells with DN-KASH. (**D**) Transition rates with reduced pulling forces. Both, in the absence of fibronectin (green) and the mechanical connection between cytoskeleton and nucleus (blue), cellular transition rates are reduced compared to WT cells on fibronectin-coated substrates (black). (**E**) Nuclear velocities with reduced pulling forces. In the pushing-dominated regime nuclear velocities are relatively unaffected by the lack of focal adhesions (green) and the transfection with DN-KASH (blue). (**F**) Quantification of cytosolic calcium levels at different degrees of confinement. The mean calcium intensity is significantly increased in cells located in narrow constrictions (first column) compared to cells in the chamber (second and fourth columns) and in wide constrictions (third column) [box plot (*n*_1_ = 20; *n*_2_ = 20; *n*_3_ = 30; *n*_4_ = 20 from two biological replicates for each condition) with whiskers extending to ±1.5 × interquartile range]. ***P < 0.001, **P < 0.01 as detected by Mann-Whitney *U* test.

The mechanical characterization of the nucleus allows us to obtain deeper insights into cellular force generation during confined migration (*40*). Mechanical forces, including pulling and pushing, are essential to deform the cell nucleus to facilitate movement into confinement (*15*, *29*, *31*, *33*). Here, we examine the changes in nuclear shape relative to their spherical shape as a tool to deduce whether the nucleus is “pulled” or “pushed” (*15*, *29*, *31*–*33*). The nuclear dimensions in the two unconfined directions are indicative of the forces applied onto the nucleus (*40*). Using 2D time-lapse microscopy data, we infer the 3D shape of the nucleus using the 2D aspect ratio (AR) and the measured nucleus Poisson ratio (Fig. 4A, inset). This allows us to compare the x-z ARs of nuclei in the channel to those of force free nuclei (section S2 and fig. S8). We introduce a dimensionless shape parameter ε =AR_confined_/AR_free_, where AR_confined_ and AR_free_ denote the confined and force free nuclear x-z ARs, respectively. Values exceeding 1 indicate that in confinement the nucleus is being stretched in the direction of migration, resulting in an even more elongated morphology than expected for an isotropic expansion under lateral compression. Values below 1 indicate that the nucleus is compressed in the migration direction, resulting in a less elongated morphology compared to the case of isotropic expansion (fig. S12). Note that, in both cases, the nucleus will still appear elongated when observed in the *x-y* plane because of the compression from the walls. As shown in Fig. 5A, in the CEM regime ε initially rises with increasing confinement width up to a maximal value of 1.4 at a channel width of 12 μm. When transitioning into the CRM regime, ε starts to decrease and eventually drops to values below 1 for channel widths below 7 μm, where it reaches a value of 0.5 at 4-μm confinement width. Thus, wild-type MDA-MB-231 cells show a nonmonotonic dependence of the shape parameters ε with channel width, suggesting a change in the forces acting on the nucleus with varying confinement.

On the basis of our characterization of the mechanical properties of the nucleus in confinement, we can relate the differences in nuclear deformations to changes in the forces that are applied to the nucleus. We assume that there are two contributions to these forces: a pulling force *F*_pull_ that is generated in front of the nucleus and a pushing force *F*_push_ acting from behind the nucleus. Overall, these forces result in a total deformation force *F*_deform_ = (*F*_pull_ − *F*_push_)/2 on the nucleus in the direction of migration (section S2 and fig. S9). If pulling forces dominate, then there is a positive deformation force in the migration direction leading to an elongated nucleus (ε > 1), while a dominant pushing contribution results in a negative value of the deformation force with a compressed nucleus (ε < 1) (see Fig. 5A, inset). Thus, our mechanical nucleus model suggests that the change in the nuclear deformation behavior with changing channel widths can be interpreted as a transition from a purely pullingbased migration regime at wider channel widths to a combined migration regime at channel widths below 7 μm with a bigger contribution from pushing forces when the nucleus is located in the center of the channel. To generate pulling forces, cells rely on a force transmission between focal adhesions at the front of the cell and the nucleus through actin fibers (*29*). To challenge our interpretation of the nuclear shape, we aim to selectively disturb this mechanism by separately targeting focal adhesions and the linkage between cytoskeleton and the nucleus. We expect that this results in lower pulling forces, which should be reflected by lower ε values. Therefore, we reduce the amount of focal adhesions by using patterns that lack fibronectin coating (see Fig. 5B). To disturb the mechanical connection between actin fibers and the nucleus, we transfect the cells with dominant-negative KASH (DN-KASH). The overexpression of DN-KASH disrupts the LINC complex by saturating available binding sites at the nuclear envelope (see Fig. 5C and fig. S5). Consistent with our expectation, in both cases, the nuclear shapes display a less elongated morphology (lower ε values) in confinement (Fig. 5A).

This inhibition of pulling forces reduces transitions over the whole range of channel widths, even in the pushing-dominated regime (see Fig. 5D). This indicates that to generate substantial pushing forces, cells first need to pull their nucleus into the constriction, deforming it in the process. Subsequently, in the constriction regime where substantial pushing forces are active, we observe a rounded, actin-enriched cortex at the rear of the cell (see fig. S12B). This observation agrees with recent findings that emphasize the presence of increased actomyosin contractility in the posterior cortex (*32*). This increased contractility results in an osmotic pushing force, which drives the nucleus through the channel. Consequently, in the pushing-dominated regime nuclear velocities in the center of the channel are only slightly reduced when the pulling mechanism is inhibited (see Fig. 5E). Recent work establishes increased Ca^2+^ levels as a signature of this mechanism (*19*, *20*). To validate this mechanism in our system, we quantify cytosolic calcium levels for different degrees of confinement (see Fig. 5F and fig. S6). Notably, when the nucleus is strongly confined, we find significantly increased calcium levels. Irrespective of the channel width, we find reduced calcium levels in cells where the nucleus is in the chamber. This suggests that calcium levels are only transiently increased while the nucleus is deformed and pushed through the constriction. To gain further insights into the extent to which increased calcium levels affect pulling and pushing forces differently, we correlate the measured calcium levels with the observed length of the nucleus (see fig. S12, C and D). This reveals a strong anticorrelation (−0.7) between nuclear length and calcium levels. Together with the observation of increased myosin activity in response to calcium release, this suggests that release of Ca^2+^ in the cytosol predominantly increases pushing forces.

Both pushing and pulling forces rely on actomyosin contractility. To investigate how the balance between pulling and pushing forces is influenced by actomyosin contractility, we treat the cells with the ROCK inhibitor Y27632 and the contractility activator Calyculin A. The multifaceted effect of these drugs renders a mechanistic interpretation of these results difficult. Nonetheless, our analysis suggests that in both Y27632-and Calyculin A–treated cells, the relationship between pulling and pushing forces shifts in favor of pushing compared to the wild type (see fig. S11).

### A mechanistic model demonstrates adaptive force generation in confinement

**Fig. 6.**
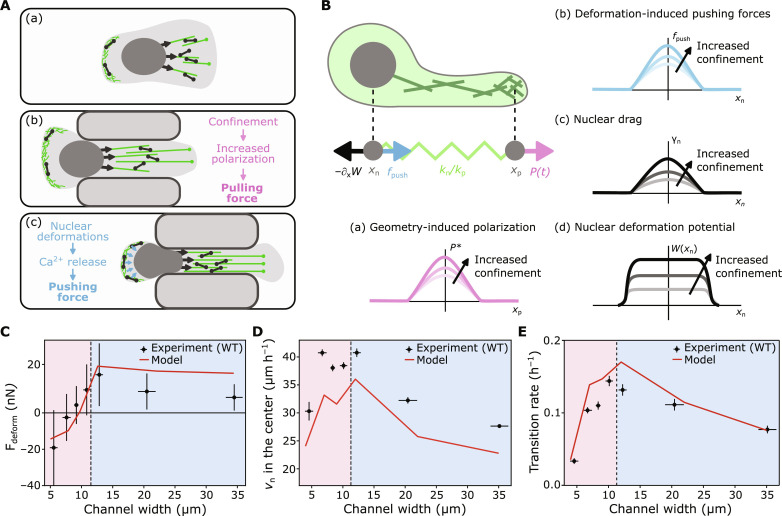
Mechanistic model for the adaptation of cellular forces to the degree of confinement. (**A**) Sketch of the proposed effect of the degree confinement on the cellular force production mechanisms. Compared to the unconfined case (a), confinement of the protrusion (b) stimulates protrusion growth resulting in an increased pulling force. Strong deformations of the nucleus (c) trigger Ca^2+^ release, which results in a higher cortical contractility. Consequently, the pressure in the rear of the cell increases, resulting in an increased pushing force onto the nucleus. (**B**) Sketch of a minimal mechanistic model for mesenchymal cell migration. The protrusion and the nucleus are coupled elastically. (a) Protrusion growth is driven by a polarization force that accounts for the internal organization of the cell and that increases in confinement. (b) In addition, when the nucleus gets deformed the cell can generate a pushing force that acts directly on the nucleus. (c) As the nucleus moves through the channel, it experiences a drag force that increases with decreasing channel width. (d) The confinement-induced deformations lead to a force that pushes the nucleus out of the channel. (**C** to **E**) Fit of the mechanistic model to key experimental observations. Error bars: (C) Error bars represent 1 SD. Error bars in (D) and (E) associated with the *y* axis represent the SE, and error bars of the channel width represent the SD.

We explore the idea of an adaptive force generation mechanism by developing a model for cellular force generation during confined migration. The key aspect of the model is the combination of two confinement-dependent force generation mechanisms: pulling forces, generated by actomyosin contractility at the front of the cell (*7*, *10*, *15*, *30*), and pushing forces (*15*, *32*, *33*), regulated by the cortical tension in the rear (Fig. 6A). We incorporate these two mechanisms by generalizing a simple dynamical model that describes mesenchymal cell migration on 2D substrates (*35*) to 3D (see section S4 for details) and including nuclear deformations in confinement. Our model consists of three degrees of freedom: the nuclear position, the position of the leading protrusion of the cell, and a polarization (Fig. 6B). The nucleus and the protrusion are coupled through an elastic spring *k*. We absorb the friction coefficients of the nucleus and the protrusion into the spring constants and denote the rescaled spring constants as *k*_n_ and *k*_p_, respectively. In previous work, we found that confinement of the protrusion can stimulate protrusion growth (*35*, *41*), which we model by including a positive selfregulation on polarization in strong confinement (Fig. 6Ba). Motivated by our data shown in Fig. 5F and recent findings in other work indicating that nuclear deformations can trigger increases in cortical tension through Ca^2+^ release (*19*, *20*) (Fig. 6Ac), we thus include an increase in pushing forces with nuclear deformation (Fig. 6Bb). Further, we account for the confinement of the nucleus by increasing the effective nuclear drag coefficient γ_n_ with decreasing channel width (Fig. 6Bc). Last, we model the effect of elastic nuclear deformations by introducing an increasing elastic energy barrier with increasing nuclear confinement (Fig. 6Bd). To allow a comparison to our experimental analysis of the nuclear shapes, we use our mechanical model for nuclear deformations to relate the pulling forces, exerted by the protrusion onto the nucleus, and the pushing forces in our model to nuclear deformations.

To constrain the free parameters of our mechanistic model, we simultaneously fit our model to a number of key experimental statistics: the deformation force acting in the direction of migration (Fig. 6C), the nuclear velocity in the center of the pattern (Fig. 6D), and the transition rate (Fig. 6E). Our model suggests the following interpretation of biphasic channel width dependence observed in both the nuclear deformations and the migration dynamics: At widths wider than the size of the nucleus (the CEM regime), stronger confinement leads to enhanced polarization and thus protrusion growth. This results in a larger force pulling on the nucleus from the front resulting in increased nuclear velocities and transition rates (Fig. 6, C to E). By contrast, for channel widths below the nuclear width (the CRM regime), increased pushing forces from the rear of the cell result in a decreasing *F*_pull_ − *F*_push_, eventually even reaching negative values (Fig. 6C). Despite the increased polarization and the additional pushing force, the increased nuclear friction result in a decrease of the predicted nuclear velocity in the channel (Fig. 6D). In addition, the increasing elastic energy barrier, associated to nucleus deformations, hinders the movement of the nucleus into the channel, resulting in a drop of the transition rates (Fig. 6E). Notably, our model indicates that while cellular pushing forces only contribute little to the overall transition rates, they strongly contribute to the observed nuclear velocities at narrow channel widths (fig. S19).

To test the predictive power of our model, after having constrained all parameters, we calculate the probability distributions of the nuclear position and velocities in our simulations at different channel widths. The probability distribution of the nuclear position transitions from a broad distribution at wide channel widths to a strongly double-peaked distribution for narrow channels, which agrees semiquantitatively with our experiments (fig. S17A). Both in the experiment and in the simulations, the distribution of nuclear velocities peaks at zero, irrespective of the channel width but decreases in spread with decreasing channel width (fig. S17, B and C). Further, our mechanistic model also allows us to connect features in the NCMs to cellular mechanisms. To do so, we compute the effective underdamped nuclear dynamics of our model (section S4). Similar to the dynamics inferred from experiments, we observe a deterministic flow from one chamber to the other with a pronounced acceleration in the channel (fig. S17D). Further, we find that our model successfully predicts most of the key features observed in the NCMs (fig. S17E): At the channel entrance, the elastic barrier associated with nuclear deformations results in a region of Δ*F_w_* < 0, followed by a pronounced region of Δ*F_w_* > 0 associated with the recoil of the contractile actomyosin structures in the protrusion and the additional pushing forces acting onto the nucleus. Last, as the cell leaves the channel, we observe a region of deceleration (Δ*F_w_* < 0) as the nucleus catches up with the protrusion. In conclusion, our mechanistic model demonstrates that cells transition from pulling to pushing dominated migration to generate sufficient deformation forces in confinement. This model not only explains the observed nuclear deformations but also allows for a mechanistic interpretation of the effective cellular dynamics inferred from experimental data.

## DISCUSSION

In summary, we studied the repeated self-imposed migration of single cells through compliant 3D channels. We found that, in the regime of wide channels, with channel width wider than the nucleus (CEM), transition frequencies and velocities increase with confinement. In contrast, migration at subnucleus confinement (CRM) is impeded by 3D confinement. A similar biphasic behavior was observed in the elastic deformation of the nucleus from oblate to prolate shapes. Overall, we find that nuclear deformations are reversible with relative volume reductions of the nucleus up to 10%. The deformation has a multifaceted impact on the cell dynamics in the channel, with marked slowing down during the entry phase as reflected in the acceleration maps obtained through the inference of nonlinear migration dynamics at varying channel widths. To explain these observations, we propose an extended dynamical cell migration model that accounts of the adaptive modulation of forces in response to confinement. The increase in pushing forces within confinement, together with elastic deformations of the nucleus and increased effective friction in the channel, explains both the observed nuclear deformations and the overall migration dynamics of the cells across a broad range of channel widths.

Our dynamical model successfully predicts key features of the NCMs, which allows for a mechanistic interpretation of the inferred dynamics in terms of confinement induced elastic nuclear deformations and cellular force adaptation. In the future, this approach could be further extended by using alternative inference approaches that account for additional degrees of freedom, allowing for the identification of other confinement adaptation mechanisms directly from data. While volume reduction of the nucleus due to the water-permeable nuclear membrane (*30*) has been hypothesized, an experimental confirmation of the compressibility so far was difficult (*28*). The observed decrease in nuclear volume within confinement, we report here, supports the notion of a purely elastic response with a distinct Poisson ratio. Previous work showed that some cell lines actively adapt their nuclear stiffness in confinement (*42*, *43*). However, by comparing the Young’s moduli of confinement-deformed and undeformed nuclei probed by AFM, we found no evidence for nuclear mechanical adaptation effects, such as nuclear softening, at the timescale of our experimental system.

Both pulling and pushing forces have been qualitatively identified to play a role in confined cell migration (*15*, *29*, *31*, *32*). Our analysis of nuclear deformations in confinement provides a quantitative measure of the balance between pulling and pushing forces, which indicates a confinement-induced adaptation of cellular force generation. A mechanism that is involved in this force adaptation is the up-regulation of cortical contractility in response to externally induced nuclear confinement (*19*, *20*), as indicated by the significant increase in cytosolic calcium levels. A recently proposed mechanism that could explain the differential impact of calcium on pushing and pulling forces could involve pressure-driven disassembly of microtubules in the rear of the cell, which gives rise to a local reinforcement of critical contractility (*33*). Here, it would be interesting to confirm this mechanism also for MDA-MB-231 cells.

We found that the pulling-dominated regime depends on focal adhesions and mechanical linkage between cytoskeleton and nucleus, which agrees with prior laser ablation experiments (*29*). In our system, reduction of pulling forces also decreases transition rates even in the pushing-dominated regime. However, once the nucleus is moved into the channel, its movement is relatively independent of the strength of the pulling force, which was also shown previously in the context of 3D confined cell migration (*32*).

Overall, our study shows that, during self-imposed confined cell migration, the nucleus can be considered as a compressible elastic object with a Poisson ratio of 0.40 ± 0.02, independent of the channel width, which is translocated by a responsive cytoskeleton that is sensitive to confinement. Previous studies (*19*, *20*) have shown that cells use their nucleus to sense and adapt to externally imposed confinement. Our work adds to this by providing evidence that the force generating cytoskeleton adapts by switching from purely pulling-based to combined pulling- and pushing-based force generation to overcome constrictions. Here, it would be interesting to study these mechanisms also in other cell lines with different mechanical properties. Increased plasticity of the nucleus could lead to memory effects, altering cellular dynamics or force generation. Our results contribute to a more comprehensive mechanistic understanding of the complex interplay between confinement, the nucleus, and the cytoskeleton during mesenchymal cell migration.

## MATERIALS AND METHODS

### Cell culture

MDA-MB-231 human breast carcinoma epithelial cells, coexpressing histone-2B mCherry (gift from T. Betz, Göttingen), were cultured in a standard common growth medium, specifically L-15 (Sigma-Aldrich), supplemented with 10% fetal bovine serum (Sigma-Aldrich). The cells are cultivated at a temperature of 37°C until reaching a confluence level of 80 to 90%. Following this, the cells were washed and trypsinized for 4 min. For experimental purposes, the cell solution was centrifuged at 1000 r.c.f. for 3 min. Following this, the cells were resuspended in L-15 medium. Approximately 20,000 cells were seeded per μ-Dish (ibidi) and allowed to adhere for a minimum duration of 3 hours. For inhibitor experiments, 0.5 nM Calyculin A (Thermo Fisher Scientific) and 30 μM Y27632 (SigmaAldrich) were added 2 hours before the start of the experiment. For live cell imaging of actin, MDA-MB-231 cells stably transduced with LifeAct GFP were used (gift from T. Betz, Göttingen).

### Plasmid vectors

pDNA Paxillin-EGFP (enhanced green fluorescent protein) was a gift from K. Rottner (TU Braunschweig). mCherry DN-KASH was a gift from D. Conway (Addgene plasmid no. 125553). pEGFP-N1 vector was purchased from Clontech. The pVAX-A120 vector and the pSTI-A120 vector are a gift from C. Rudolph (ethris GmbH).

To generate the desired vector, the Gibson DNA assembly (GDA) reaction was conducted by using the NEBuilder HiFi DNA Assembly kit from NEB (NEB, NEBuilder Hifi DNA Assembly Cloning Kit, E5520S) as described. In the first step, we generated a pVAX-KASHeGFP vector by assembling KASH from the mCherry DN-KASH vector and EGFP from the pSTI-A120 vector into the pVAX-A120vector. Briefly, the coding region for KASH and the coding region from EGFP was polymerase chain reaction (PCR) amplified using the Q5 High-Fidelity PCR Kit (NEB, E0555S). All oligonucleotide primers were designed so that the DNA fragments to be assembled overlap each other by at least 25 bp at the ends. Sequences of all primers are provided (table S4). The assembly reaction contained approximately 10 pmol of insert DNA and 2 pmol of the Nhe I/Eco RI–linearized pVAX-A120-vector and was incubated at 50°C for 15 min. After the GDA reaction was completed, the reaction mix was transformed into chemically competent *Escherichia coli* (*E. coli*) cells (NEB, NEB no. C2987). The transformed LB (lysogeny broth)–*E. coli* mix (100 μl) was plated onto LB/kanamycin plates, followed by 37°C incubation overnight. Single colonies were picked, and positive clones were further verified by DNA sequencing. One clone was chosen for the next cloning step to generate the pDNA-DN-KASH-EGFP. Briefly, the coding region for KASH-EGFP was PCR amplified using the Q5 High-Fidelity PCR Kit (NEB, E0555S). The oligonucleotide primers for the KASH-EGFP-fragment were designed so that the DNA fragments to be assembled overlap each other by at least 26 bp at the ends (table S4). The assembly reaction contained approximately 60 pmol of insert DNA and 20 pmol of the Age L/Not L-linearized mCherry DN-KASH vector and was incubated at 50°C for 15 min. After the GDA reaction was completed, the reaction mix was transformed into chemically competent *E. coli* cells. The transformed LB–*E. coli* mix (100 μl) were plated onto LB/kanamycin plates, followed by 37°C incubation overnight. Single colonies were picked, and positive clones were further verified by DNA sequencing.

### Transfection of MDA-MB-231 cells

MDA-MB-231 m-Cherry cells were seeded in a six-well plate with an initial confluency ranging from 70 to 80%. Transfection was performed using a commercial transfection reagent, *Trans*IT-BrCa (Mirus), at a dose of 1 μg of pDNA per well, with a *Trans*IT-BrCa–to–pDNA ratio of 2.0 μl of *Trans*IT-BrCa per 1 μg of pDNA, following the manufacturer’s instructions. Briefly, pDNA was diluted in OptiMEM to a total volume of 200 μl, followed by the addition of *Trans*IT-BrCa to the pDNA-OptiMEM solution. The mixture was incubated for 30 min at room temperature and then added dropwise to the cells in complete growth media. The cells were incubated with the TransIT-BrCa Reagent:DNA complexes for 24 hours at 37°C in total transfection volume of 2.5 ml. Subsequently, the transfected cells were detached and seeded into the hydrogel-based dumbbell structures. Sequences of both pDNAs used are provided (table S4).

### Preparation of dumbbell-shaped hydrogel cavities

The experimental procedure uses a μ-Dish ibiTreat (ibidi). To establish a defined height for the hydrogel layer containing the desired structures, PDMS stamps were used (see fig. S1). The PDMS, in a 10:1 monomer cross-linker ratio, was poured onto a specific silicon wafer, subjected to degassing, and left to cure overnight at 50°C. PDMS stamps containing small square pillars (200 μm by 200 μm by 20 μm) underwent activation for 3 min in an ultraviolet (UV) cleaner (PSD-UV, Novascan). After activation, the stamps were placed onto the μ-Dish. A droplet of PEG-NB hydrogel precursor was put adjacent to the stamps and drawn into the free space provided by the small pillars of the PDMS stamp (additional details on the hydrogel composition can be found in section S1.1). The hydrogel precursor was then selectively photopatterned using the PRIMO module (Alvéole) integrated into an automated inverted microscope (Nikon Eclipse Ti). Design specifications for the desired hydrogel structures were generated using vector-based software (Inkscape, see exemplary design in fig. S1Ac), transferred to Leonardo software (Alvéole), and subsequently polymerized upon exposure to UV light (365 nm) at a dose of 3 mJ/mm^2^, according to the transferred drawing. Following illumination, the stamps were removed, and the dish was thoroughly rinsed with milliQ water, followed by rehydration with phosphate-buffered saline (PBS) for 5 min. Subsequently, the μ-Dish was incubated with a human fibronectin (YO Proteins) solution (50 μg ml^−1^) for 45 min. The ECM protein binds exclusively to the bottom of the dumbbell structures, composed of the μ-Dish substrate, owing to the passivating attributes of the PEG-NB hydrogel. Following the incubation period, the μ-Dish was rinsed three times with PBS. After a 45-min interval, the μ-Dish was rinsed again with PBS and was then stored at room temperature in PBS until cells were seeded onto it.

### Microscopy and cell tracking

Measurements were performed in time-lapse mode for up to 40 hours on a Nikon-Eclipse Ti-E inverted microscope. To ensure consistent incubation conditions throughout the measurements, the microscope was equipped with gas incubation and a heating system (Okolab). Bright-field and fluorescence images of the fibronectin-coated pattern and the coexpressed labeled histones were acquired every 10 min. To enhance the quality of the nuclei images, a band-pass filter was applied. Following this, the images were binarized, and the positions of the nuclei’s center of mass were determined using the Analyze Particles plugin in ImageJ.

### Nuclear volume analysis

To analyze the changes in nuclear volume over time, z-stacks within the frame of a time-lapse recording over 20-hour z-stacks of MDA-MB-231 cells transmigrating through the constriction were captured with a confocal microscope (LSM-980, Zeiss). To provide standard incubation conditions throughout the measurements, the microscope was equipped with gas incubation and a heating system (Okolab). The nuclear volume for each timestamp was analyzed by the software “arivis Vision4D” (Zeiss) (see fig. S2). For further details, see section S1.

### Stiffness evaluation via AFM

AFM was used to assess mechanical properties, including the Young’s modulus, of both the hydrogel and the cell nuclei. The measurements were conducted using a JPK NanoWizard II (JPK Instruments), which was interfaced with an inverted optical microscope (Zeiss, Axiovert 200 M). The used cantilevers were modified with beads, having diameters of either 3.5 μm (NanoAndMore GmbH; type: CP-PNPL-SiO-B, nominal spring constants of 0.08 N/m) or 3.6 μm (sQUBE, type: CP-CONT-PS-B, nominal force constants between 0.02 and 0.77 N/m). To calibrate the cantilever sensitivity, the slope of the force-distance curve against the hard petri dish substrate was recorded. Subsequently, the spring constants of the cantilevers were measured using the thermal noise method provided by the AFM software (JPK SPM). The Young’s modulus of the hydrogel was determined by applying large forces (>10 nN) through the bead, resulting in indentation depths of up to 2 μm. Likewise, the stiffness of the cell nuclei was determined using the same atomic force spectroscopy method by applying force (5 nN) to the cell nuclei (see fig. S3B). Typically, a square grid consisting of 64 points with 1-μm spacing between neighbors was tested at a single position to ensure statistically robust results. Force-distance curves were obtained with an extension speed of 2.5 μm/s. The Young’s moduli of the tested objects were extracted by fitting the force-distance curves to a Hertz contact model.

### Bead displacement analysis

The tracking of fluorescent nanobeads embedded within the hydrogel and the cell nuclei was carried out using the TrackPy Python package. To observe the movement of a particle, its displacement relative to a neutral reference position was calculated. For quantitative analysis of the observable deformation in the gel, the tracking algorithm was used to trace the positions of at least 20 embedded fluorescent nanobeads in each experiment. To visualize their displacement, the difference between the positions of the marker beads at each time step and their initial neutral position was determined. Considering that the marker beads are embedded within the gel, this is due to their larger diameter of 200 nm compared to the approximate mesh size of the hydrogel, which is approximately 40 nm. Consequently, the displacement field exhibited by the beads reflects the deformation field of the surrounding hydrogel. For further details, see section S1.

### Nuclear shape analysis

To quantify the effect that external forces have on the confined nucleus in the center of the channel, we computed the deviation from an isotropic (force free) expansion of the nucleus under compression through the channel walls. To find an approximate expression for the dimensions of the nucleus under compression in the presence of pulling and pushing forces acting along the *x*–direction, we first calculated the shape of the unconfined nucleus with pulling and pushing forces applied and then applied the confinement-induced deformation in the *y*-direction together with an isotropic expansion in the *x*/*z*-direction. For further details, see section S2.

### Calcium level analysis

Cytosolic calcium levels were quantified across varying degrees of spatial confinement using the calcium indicator Calbryte (aat bioquest). Experimental procedures involved the creation of dumbbell-shaped cavities, cell seeding, and subsequent application of Calbryte520 AM at a concentration of 10 μM after a 2-hour incubation period. Following a 45-min incubation with the calcium indicator, dishes were gently rinsed before imaging using a fluorescence microscope. Calcium levels within the cytosol were assessed by integrating the fluorescent signal across the entire cytosol. For further experimental details, see section S1.11.

## Supplementary Materials

### This PDF file includes:

Supplementary Text Figs. S1 to S19 Tables S1 to S3 Legend for table S4 Legends for movies S1 to S8 References
