# Supplementary material for: Nuclear deformation and dynamics of migrating cells in 3D confinement reveal adaptation of pulling and pushing forces
Source: Sci Adv. 2024 Aug 21;10(34):eadm9195. doi: 10.1126/sciadv.adm9195 (PMC11338266; doi:10.1126/sciadv.adm9195)
Supplement: Supplementary file 1 — Supplementary Text Figs. S1 to S19 Tables S1 to S3 Legend for table S4 Legends for movies S1 to S8 References [file sciadv.adm9195_sm.pdf]

Science Advances
AAAS

Supplementary Materials for

Nuclear deformation and dynamics of migrating cells in 3D confinement reveal adaptation of pulling and pushing forces

Stefan Stöberl *et al.*

Corresponding author: Chase P. Broedersz, c.p.broedersz@vu.nl; Joachim O. Rädler, raedler@lmu.de

Sci. Adv. **10**, eadm9195 (2024)
DOI: 10.1126/sciadv.adm9195

The PDF file includes:

Supplementary Text
Figs. S1 to S19
Tables S1 to S3
Legend for table S4
Legends for movies S1 to S8
References

Other Supplementary Material for this manuscript includes the following:

Table S4
Movies S1 to S8

1. Further experimental details

1.1. Hydrogel precursor

The precursor solution for hydrogel formation was prepared using phosphate-buffered saline (PBS) containing 5 mM of 20 kDa 8-armed PEG-norbornene (PEG-NB, JenKem Technology). To enable crosslinking, an off-stoichiometric amount (0.4 – 0.8) of 1 kDa PEG-dithiol (Sigma-Aldrich), was added along with 3 mM of the photo-initiator lithium phenyl-2,4,6-trimethylbenzoylphosphinate (LAP, Sigma-Aldrich). The inclusion of the PEG-dithiol cross-linker and LAP photo-initiator enables the formation of a stable crosslinked network within the hydrogel. The mechanical properties of the resulting hydrogel can be directly influenced by varying the quantity of Peg-NB monomers and/or the amount of cross-linker. The cross-link ratio r_c is defined as the ratio of functional groups present in the cross-linker (two thiol groups per cross-linker) to the functional groups present in the PEG-NB monomer (eight norbornene groups per monomer).

$$r_c = \frac{2c(\text{dithiol})}{8c(\text{PEG-NB})} \tag{S1}$$

Fluorescent nano-beads, characterized by a size of 0.2 µm, were incorporated into the precursor solution with a total concentration of 5.8E+08 ml^{-1} (Distrilab). These embedded nano-beads function as force sensors due to the elastic properties of the hydrogel.

1.2. Photolithography

Silicon wafers undergo a series of treatment steps to prepare them for further processing. Initially, the wafers are subjected to hydrofluoric acid treatment (TECNIC) to clean the surface. Following this, a layer of photoresist AZ40XT (AZ Electronic Materials) is spin coated onto the wafer, resulting in a thickness of approximately 20 µm. The coated wafer is then subjected to a soft bake process. To achieve the desired patterns, laser direct imaging (Protolaser, LPKF) is used. Following exposure, a post-exposure bake step is performed to further stabilize the resist. Subsequently, the wafer undergoes development using AZ 726 MIF (AZ Electronic Materials) to remove the unexposed photoresist, revealing the patterned geometries. In the final step, the wafer surface is treated with (Trichloro(1H,1H,2H,2H-perfluoro-octyl)silane (Sigma-Aldrich) in a process known as silanization. This treatment imparts a hydrophobic character to the wafer surface by bonding the silane molecules, which helps control surface interactions in subsequent processes.

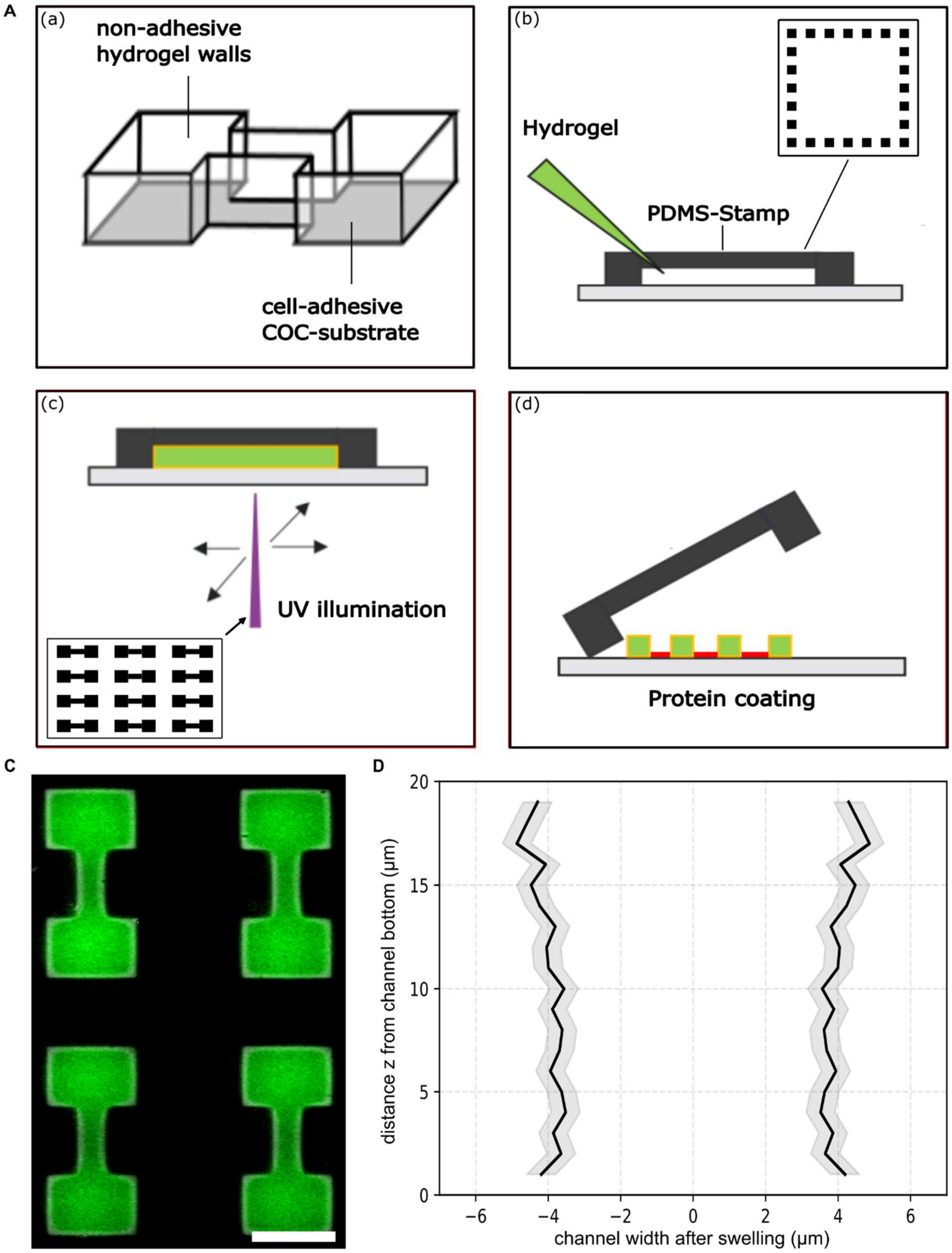

Fig. S1. Validation of the experimental specifics. (A) Step-by-step protocol for fabricating hydrogel-dumbbell cavities. (**B)** Determination of the characteristic dimensions of the dumbbell structures, with the bridge width serving as the variable parameter. **(C)** Illustration of the fibronectin-coated bottom surface of the dumbbell structures (scale bar, 40 um). **(D)** Analysis of the correlation between the constriction width and the spatial distance *z* from the bottom of the channel.

Cell line	Bridge width (µm)	Cells	Transitions	Timepoints	Biol. Replicates
MDA-MB-231 wildtype	4.6 ± 0.7	144	82	16468	14
	6.7 ± 0.7	333	639	41127	16
	8.4 ± 0.4	211	434	26256	16
	10.1 ± 0.6	164	449	20770	16
	12.2 ± 0.6	102	279	13939	6
	20.4 ± 1.5	99	206	12351	4
	35.1 ± 1.4	249	318	27760	4
MDA-MB-231 w/o FN	7.1 ± 0.6	24	28	3725	4
	8.6 ± 0.3	43	37	6324	4
	10.0 ± 0.6	46	61	5695	4
	12.8 ± 0.8	52	38	6999	4
	15.0 ± 0.8	117	131	15957	4
	19.4 ± 0.8	45	72	6096	2
MDA-MB-231 DN-KASH	8 ± 2	50	60	8063	6
	13 ± 2	31	56	4796	6
	17 ± 2	31	26	4495	6
	20.8 ± 0.4	13	19	2328	3
MDA-MB-231 Collagen	8 ± 1	101	450	14262	6
	14 ± 2	32	150	4000	6
	15.9 ± 0.7	35	161	5273	3
	24 ± 2	45	99	6123	3
MDA-MB-231 Laminin	7 ± 2	62	200	9747	6
	12 ± 2	61	248	9184	6
	16 ± 2	8	35	1282	6
	22 ± 2	59	103	8121	3

MDA-MB-231 Calyculin A	6.8 ± 0.8	41	67	6007	2
	8.4 ± 0.3	29	57	3439	4
	10.2 ± 0.6	23	75	2672	4
	12.1 ± 0.7	12	37	1535	2
	19.2 ± 1.1	41	29	7005	2
MDA-MB-231 Y27632	4.3 ± 0.9	125	9	18101	10
	6.6 ± 0.7	237	85	30516	10
	8.5 ± 0.4	142	73	16333	8
	10.3 ± 0.7	115	98	14197	6
	12.1 ± 0.5	109	104	14144	4
	16.0 ± 0.9	153	133	20006	6
	19.7 ± 0.9	49	55	7005	4

Table S1. Bridge widths *w* of the micro-cavities and number of statistics collected. Number of cell trajectories and number of transitions recorded for each bridge width *w*.

1.3. Microstructure design

All dumbbell-shaped cavities were specifically designed to feature two islands of equal size ($(37.5 \pm 0.9)^2$ µm^2) connected by a bridge of uniform length ((35.0 ± 1.0) µm). The width of the bridge denotes as *w*, and the corresponding number of cell trajectories for each value of *w* are presented in table S1. The micropattern without constriction was designed to possess a similar width and total length as the two-state pattern with L = 35 µm, and thus has dimensions ((109.0 ± 2.0) µm) x (37.0 ± 1.5) µm).

1.4. Cell exclusion criteria

We track the position of many cells to determine the transition statistics of cells migrating through constrictions with varying bridge width. To limit the effects of ambiguous or abnormal migration behaviour, we apply the following inclusion criteria in the analysis of migration in our hydrogel micro-cavities.

1. Only a single cell occupies the micro pattern. Trajectories are cut when the cell rounds up for division.
2. The cell and its protrusions are entirely confined within the borders of the microstructure.
3. Throughout the entirety of the experiment, the cell shows no abnormalities such as the presence of multiple nuclei, cell death, or detachment from the substrate.
4. Cell dynamic statistics are included when a protrusion forms and extends to the second adhesion-site, even if the cell is unable to transmigrate through the constriction.

Criteria 1-3 represent fundamental conditions for single cell experiments within micro-cavities, and these criteria alone are applied to cells undergoing migration in cavities without constriction. Criterion 4, however, is exclusive to our dumbbell-shaped hydrogel cavities, as the migratory potential of cells within these structures is heavily influenced by the width of the constricted region, particularly in terms of physical confinement. Despite the inability of cells to transmigrate through these narrow constrictions, we consider these cells in our analysis, as such behaviour is deemed unremarkable in this context.

1.5. Nuclear growth analysis

For nuclear volume analysis, data were imported into the Arivis Vision 4D software. Subsequently, each dataset underwent initial bleach correction. Following this correction, individual nuclei were segmented and size-filtered to eliminate extraneous noise components. The segmented objects were subjected to manual proofreading, and parameters were adjusted as required, enabling the calculation of nuclear volume within the software.

To investigate the overall trend of increasing nuclear volume over time (Fig. 4A), we performed control experiments of MDA-MB-231 cells caught within a big sized hydrogel cavity without constriction ($(50.0 \pm 2.0)^2$ µm²) (fig. S2A). The evaluation of the nuclear volumes reveals no significant differences in the nuclear growth rate between the confined and unconfined conditions (mean ± SD for n = 3 cell nuclei per condition). Thus, the increasing nuclear volume over time originates from the cell growth.

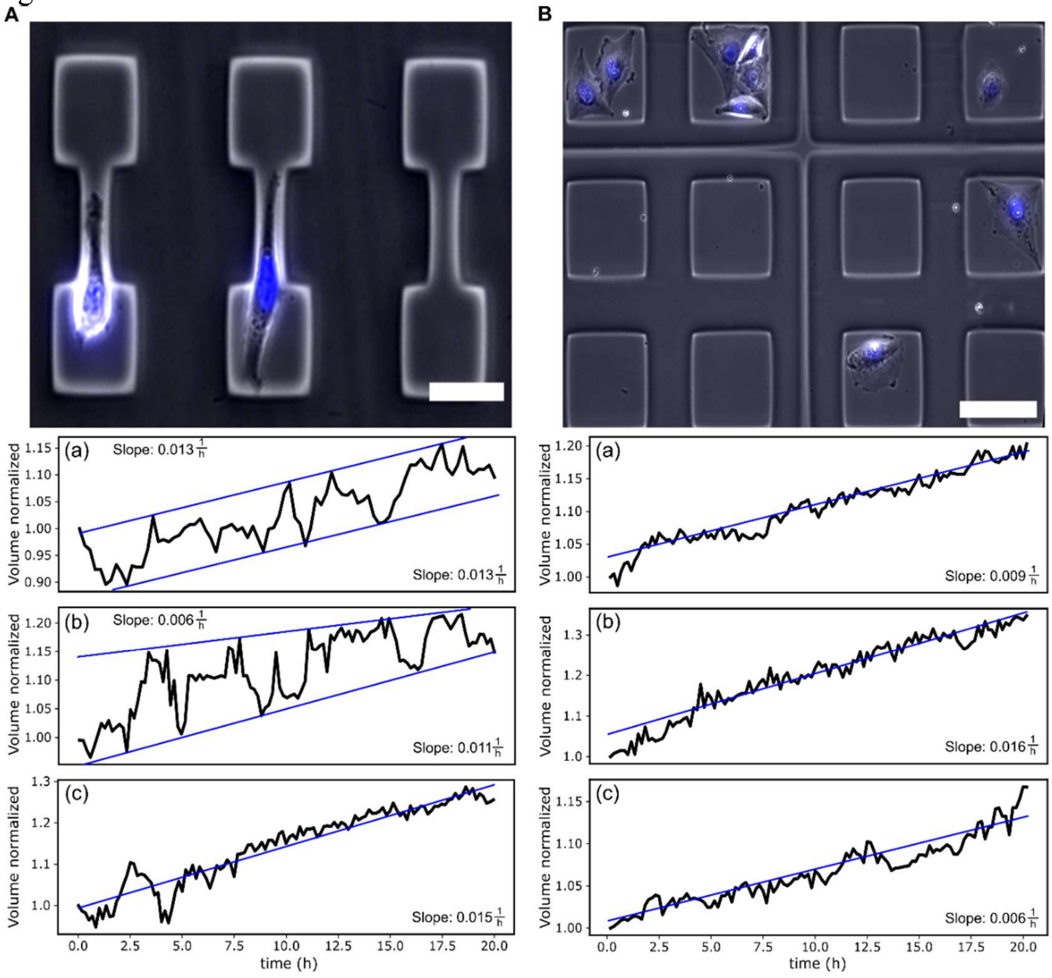

Fig. S2. Analysis of nuclear volume alterations over time. (A) Cells located within dumbbell-shaped cavities. The normalized cell volume exhibits significant variations depending on the nuclear position within the dumbbell structure (scale bar, 40 µm). Exemplary trajectories of nuclear volume for cells located within dumbbell structures are depicted in (a) – (c). **(B)** Cell nuclei positioned within hydrogel cavities where cells do not encounter constrictions. The normalized cell volume exhibits progressive growth over time (scale bar, 50 µm). Images (a) – (c) illustrate exemplary trajectories of nuclear volume for cells located within hydrogel cavities without constrictions.

1.6. Determination of the Poisson ratio

The change in volume of a material under compression can be characterized in terms of the Poisson ratio ν. A value of $\nu = 0.5$ characterizes an incompressible material, while values below 0.5 indicate that the material's volume changes under compression. To determine the Poisson ratio of the cell nucleus ν_n, we consider the changes in volume and width of nuclei entering the constriction. The Poisson ratio ν_n is given by (*44*)

$$\nu_n = \left(\frac{1}{2} - \frac{\Delta V\, W}{2V\, \Delta W}\right). \tag{S2}$$

With the following derivatives of ν with respect to $V_{rel} = \frac{\Delta V}{V}$ and $W_{rel} = \frac{\Delta W}{W}$

$$\frac{d\nu_n}{dV_{rel}} = \left(-\frac{1}{2\, W_{rel}}\right) \tag{S3}$$

and

$$\frac{d\nu_n}{dW_{rel}} = \left(\frac{V_{rel}}{2\, W_{rel}^2}\right), \tag{S4}$$

we then calculate the standard deviation (*SD*) of ν_n, which is given by

$$SD_{\nu_n} = \sqrt{\left(\frac{V_{rel}}{2\, W_{rel}^2}\, SD_{W_{rel}}\right)^2 + \left(-\frac{1}{2\, W_{rel}}\, SD_{V_{rel}}\right)^2}. \tag{S5}$$

1.7. Stiffness evaluation via AFM

AFM experiments have been performed to measure the stiffness of the hydrogel and the cell nuclei. The median of the Young's modulus for the soft hydrogel was measured at 2.9 kPa over 1465 positions in more than 20 different samples and the hard hydrogel was measured at 24 kPa over 256 positions in four different samples.

To determine the nuclear stiffness of MDA-MB-231 cells, cells were adhered on a petri dish (µ-Dish ibiTreat, ibidi) without any confinement. The median of the Young's modulus for the cell nuclei was measured at 368 Pa. These measurements include 1920 different positions on 20 different cells. The Young's modulus of the MDA-MB-231 cells agrees well with literature values(*45*, *46*).

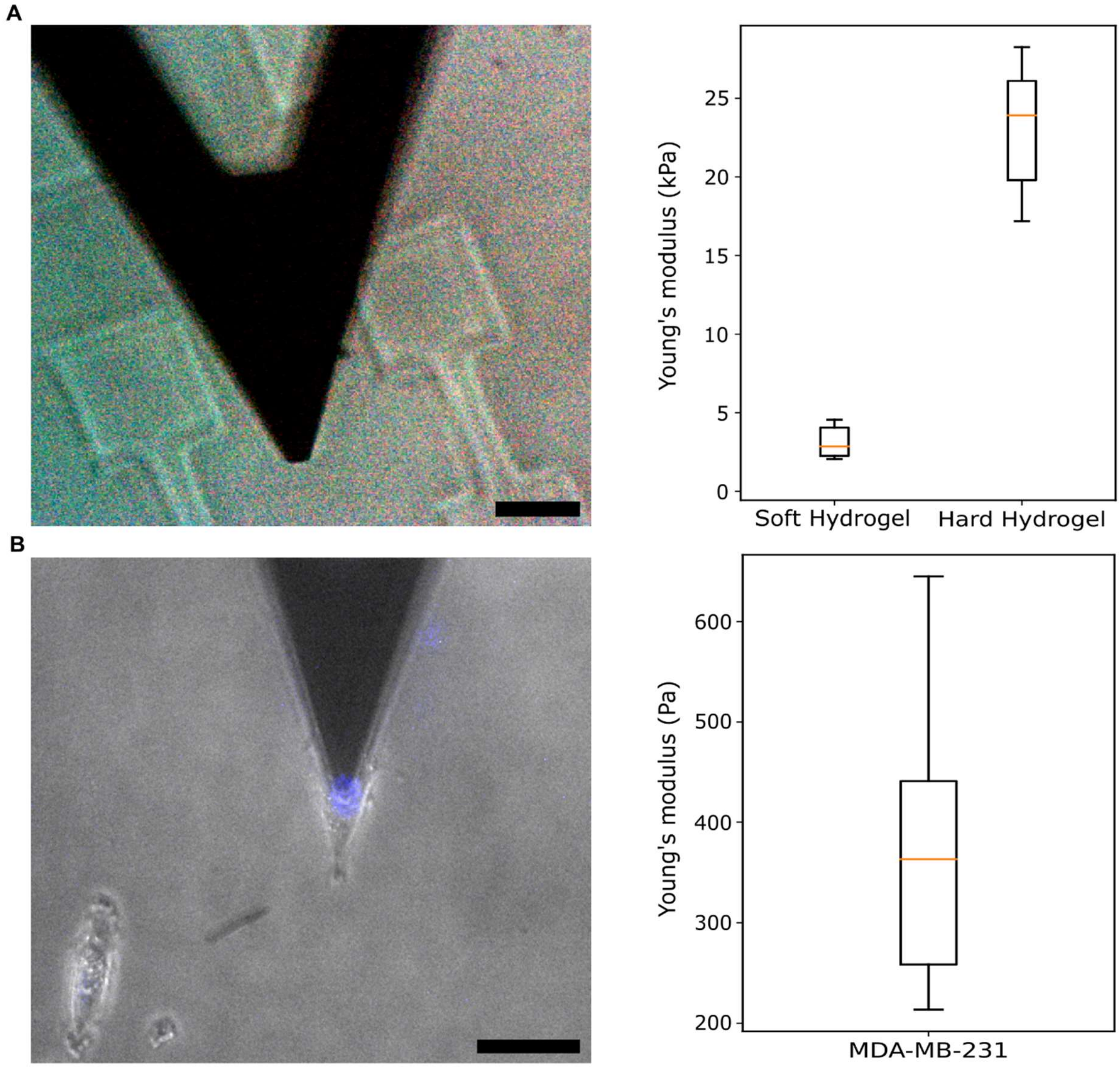

Fig. S3. Stiffness measurements of hydrogel and cell nuclei performed with an AFM. (A) Evaluation of hydrogel stiffness by measuring the interaction between two dumbbell structures, considering various compositions of the hydrogel (Box plot (n_{soft} = 1465; n_{hard} = 256) with whiskers extending to ± 1.5 × IQR) (scale bar, 20 µm.) **(B)** Stiffness measurement of the nuclei in MDA-MB-231 cells (Box plot (n = 1920) with whiskers extending to ± 1.5 × IQR) (scale bar, 20 µm).

1.8. Measured normal forces exerted by the cell nucleus

In order to evaluate the measured normal forces exerted by the nucleus, a classical Hertz' model for the gel deformation was assumed on the basis of the displacement of the fluorescent nano-beads that follow the deformation of the hydrogel. The hydrogel pushes back against an indenting sphere with the Hertz' force, according to the following expression (*47*, *48*):

$$f_h(\delta) = \frac{4}{3} \frac{E_h}{1 - {\nu_h}^2} \sqrt{R_i}\, \delta^{3/2} \tag{S6}$$

Here, δ describes the indentation into the hydrogel wall, and R_i the initial radius of the (unsqueezed) cell nucleus. E_h and ν_h are the Young's modulus and Poisson's ratio of the hydrogel, respectively.

1.9. Analysis of bead displacement orientation

Analyzing the hydrogel's motion when the cell nucleus is completely enclosed within the channel, with particular focus on the orientation of the arrows, provide valuable insights into the influence of friction within our system. The angle was determined by considering the y- and x-values of the displacement arrow.

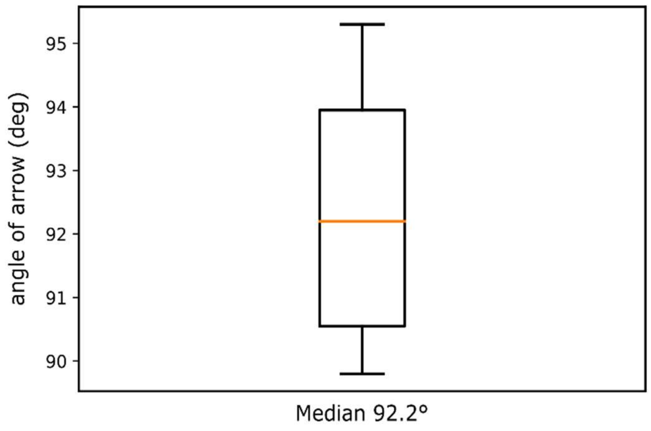

Fig. S4. Angle of bead displacement: The median value of displacement along both the vertical and horizontal axes relative to the hydrogel wall surface (Box plot (n = 500) with whiskers extending to ± 1.5 × IQR).

1.10. Transfection of MDA-MB-231 cells with DN-KASH

To perturb the pulling mechanism of cells, we transfected MDA-MB-231 cells with a GFP-labelled DN-KASH construct. The transfection with DN-KASH disrupts the LINC complex due to expression of dominant negative KASH protein.

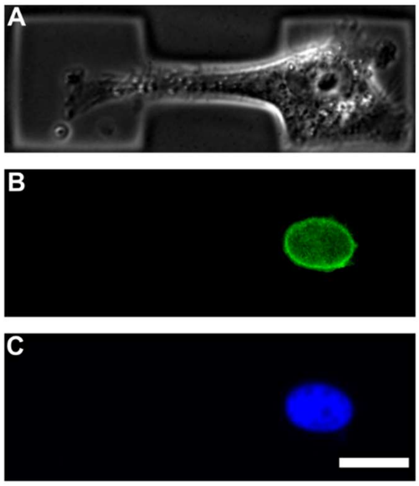

Fig. S5. DN-KASH transfection: (A) A DN-KASH transfected MDA-MB-231 cell located within the dumbbell-shaped cavity. (B) The GFP-labelled DN-KASH is accumulated at the nuclear membrane. (C) The mCherry-labelled nucleus (scale bar, 20 μm).

1.11. Quantification of the cytosolic calcium levels

Analyzing the calcium levels for different degrees of confinement of the dumbbell-shaped cavities provide valuable insights into the role of calcium in the pushing mechanism of cells to overcome narrow confinement. Therefore, we utilized the calcium indicator Calbryte520 AM (aatbio). Once Calbryte520 AM enters the cell, it is readily hydrolyzed by intracellular esterase where it becomes activated and responsive to calcium. The activated indicator is now a polar molecule that is incapable of freely diffusing through the cell membrane, essentially trapping it inside the cell. Upon binding calcium ions, Calbryte520 AM produces a bright fluorescence signal with high signal/background ratio. These experiments were performed by casting dumbbell shaped cavities with different degrees of confinement. Cells were seeded onto these structures and waited to adhere for two hours. The calcium indicator Calbryte520 AM was added with a final concentration of 10 µM, incubated for 45min, before gently rinsed with Hanks Buffer followed by adding cell culture medium L-15. To analyze the calcium levels of the cells, we integrated the calcium signal over the whole cytosol (see fig. S6B) and built the mean intensity per pixel.

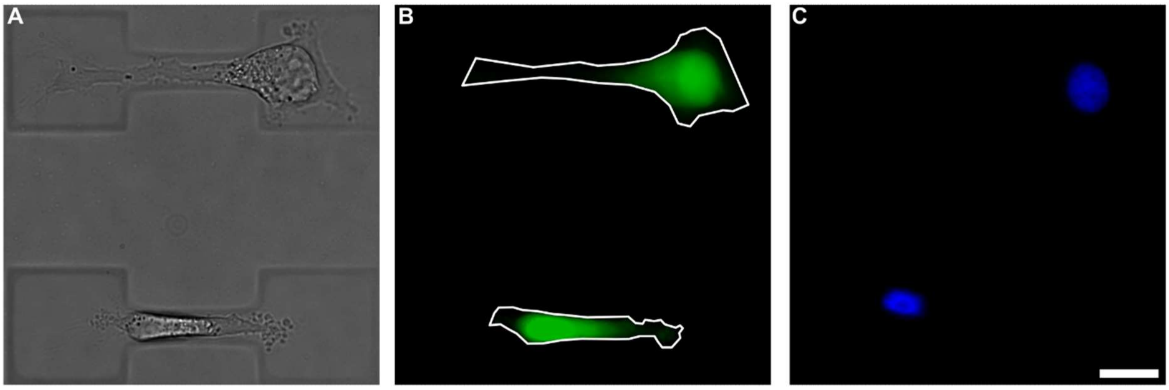

Fig. S6. Quantification of calcium levels: (A) MDA-MB-231 cells located within the dumbbell-shaped cavities. (B) The lower cell is A DN-KASH transfected MDA-MB-231 cell located within the dumbbell-shaped cavity. (B) The GFP-labeled DN-KASH is accumulated at the nuclear membrane. (C) The mcherry-labeled nucleus (scale bar, 20 µm).

2.Nuclear shape analysis

2.1. Theoretical model for the normal forces

To calculate the expected indentation of the hydrogel by a passing cell, we use a simple Hertz model, as a soft sphere is compressed in between two soft half-spaces (fig. S7). As the sphere and constricting material are in equilibrium, one can assume that the hydrogel wall is exposed to a force similar to that of a hard sphere (fig. S7A) and that at the same time, the nucleus behaves like a sphere that is squeezed between similarly infinitely hard walls (fig. S7B).

In conclusion, the force exerted by the nucleus squeezed by a length of α is given by the following equation (*48*):

$$f_n(\alpha) = \frac{4}{3} \frac{E_n}{1 - {\nu_n}^2} \sqrt{R}\, \alpha^{3/2} \tag{S7}$$

And the force that the hydrogel pushes back with against a sphere indenting δ into it, F_h is given by Equation (S6). Here, R_i describes the initial radius of the cell nucleus (unsqueezed), and R is the actual radius when squeezed. Where these two forces are equal is the equilibrium of the system and yields the expected displacement. To find this equilibrium, we write the indentation of the hydrogel as $\delta = \left(\frac{h}{2} - \frac{h_i}{2}\right)^{3/2}$ and the length of the squeezed nucleus as $\alpha = \left(R_i - \frac{h}{2}\right)^{3/2}$. Taken together and equating the two forces (fig. S7C and D) we get the following equation:

$$\frac{4}{3}\frac{E_n}{1-\nu_n^2}\sqrt{R_i}\left(R_i - \frac{h}{2}\right)^{3/2} = \frac{\left(\frac{E_n}{1-\nu_n^2}\right)^{\frac{2}{3}}}{\left(\frac{E_h}{1-\nu_h^2}\right)^{\frac{2}{3}} + \left(\frac{E_n}{1-\nu_n^2}\right)^{\frac{2}{3}}}\left(R_i - \frac{h_i}{2}\right) \qquad \text{(S8)}$$

We consider the mechanical properties of the hydrogel and nucleus, respectively. The Young's modulus and Poisson's ratio of the hydrogel are denoted as E_h and ν_h, while the Young's modulus and Poisson's ratio of the nucleus are denoted as E_n and ν_n. The initial height of the constriction, represented as h_i, refers to the distance between the hydrogel walls when no cell is present. Similarly, the initial radius of the cell nucleus, denoted as R_i, represents its size when it is not subjected to a constriction.

Physically, equation (S8) describes the following scenario: If the nucleus is compressed by a channel that is narrower than the unconfined nuclear width, the nucleus pushes outwards and deforms the surrounding hydrogel walls. As it does so the normal force generated by the confined nucleus decreases as the deformed channel width increases (red line in fig. S5D), while the counteracting force generated by the deformed hydrogel walls increases (grey lines in fig. S5D). The deformed channel width at which these two forces are equal then determines the equilibrium deformation of the channel. This corresponding equilibrium force decreases with increasing initial, undeformed channel width, resulting in the theoretical curve shown in fig. 4C in the main text that describes the equilibrium normal force as a function of the undeformed channel width.

When a cell is located within the constriction, the distance between the walls h or the diameter of the nucleus changes. If the hydrogel and the nucleus have similar stiffness properties, and the constriction width is sufficiently small, a significant difference between the initial height h_i and the final height h can be observed. This difference occurs due to the displacement of the hydrogel caused by a displacement value δ. The displacement can be calculated by solving the following equation.

$$\delta = \frac{h}{2} - \frac{h_i}{2} = \frac{\left(\frac{E_n}{1-\nu_n^2}\right)^{\frac{2}{3}}}{\left(\frac{E_h}{1-\nu_h^2}\right)^{\frac{2}{3}} + \left(\frac{E_n}{1-\nu_n^2}\right)^{\frac{2}{3}}}\left(R_i - \frac{h_i}{2}\right) \qquad \text{(S9)}$$

This equation describes the expected deformation of the hydrogel when a cell nucleus is squeezed inside a hydrogel constriction.

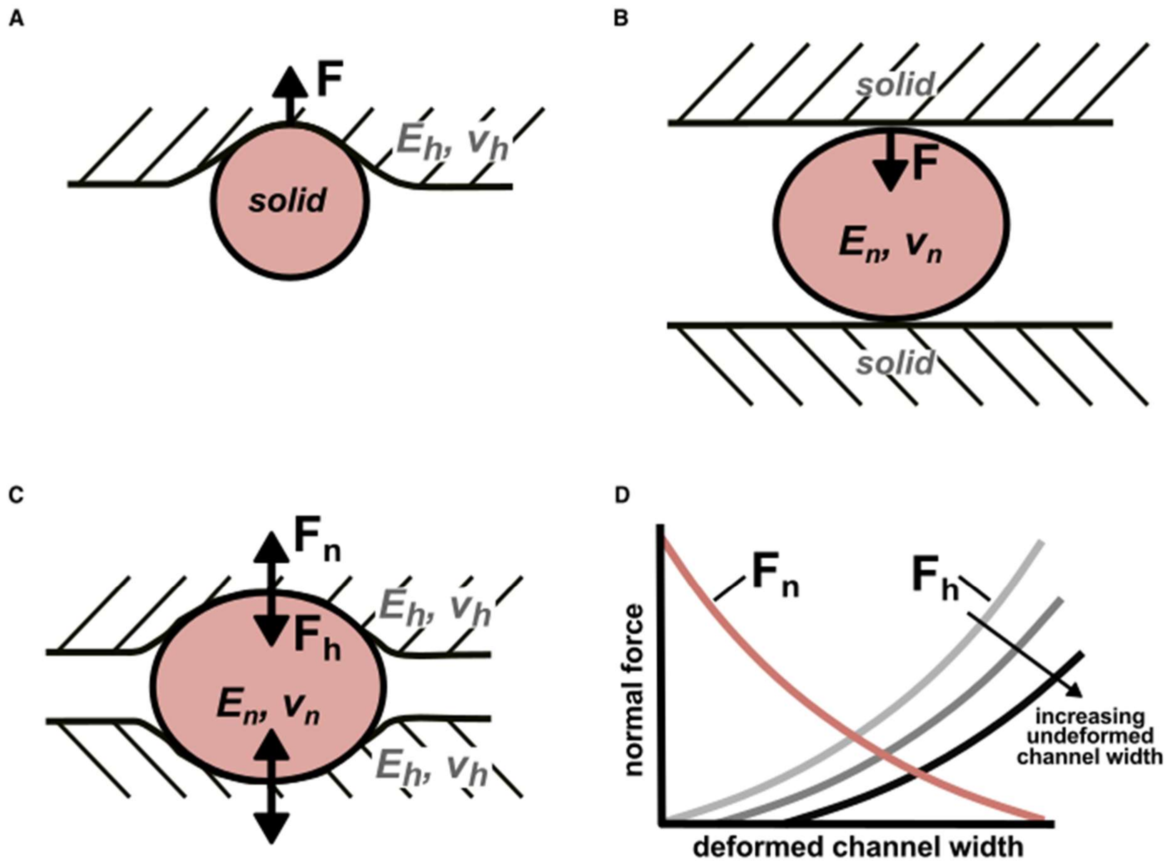

Fig. S7. Illustrations of the relevant contact mechanics setups. (A) A solid ball is pushed into an elastic half-space. **(B)** An elastic sphere experiences compression when confined between two rigid boundaries. **(C)** A deformable undergoes squeezing between two elastic half-spaces. **(D)** An illustrative depiction demonstrating the anticipated state of equilibrium between a soft sphere and a flexible half-space.

2.2. Calculation of the shape parameter

To quantify the effect that external forces have on the confined nucleus in the centre of the channel, we compute the deviation from an isotropic (force free) expansion of the nucleus under compression through the channel walls. To find an approximate expression for the dimensions of the nucleus under compression in the presence of pulling and pushing forces acting along the x-direction (fig. S8), we first calculate the shape of the unconfined nucleus with pulling and pushing forces applied and then apply the confinement-induced deformation in the y-direction together with an isotropic expansion in the x /z-direction.

The sum of pushing and pulling forces induce a strain u_{xx} in the x-direction. The strains in the orthogonal directions are then given by (*49*) $u_{yy} = -\nu_n u_{xx}$ and $u_{zz} = -\nu_n u_{xx}$. For small deformations along the x-direction, the strains can be written as (*50*) $u_{xx} = \frac{dx}{x}$, $u_{yy} = \frac{dy}{y}$, and $u_{zz} = \frac{dz}{z}$. To find deformations Δy and Δz induced by the forces acting in x–direction, we integrate the infinitesimal strains and use that $u_{yy} = u_{zz} = -\nu_n u_{xx}$, such that

$$\int_{y_0}^{y_0+\Delta y} \frac{dy}{y} = \int_{z_0}^{z_0+\Delta z} \frac{dz}{z} = -\nu_n \int_{x_0}^{x_0+\Delta x} \frac{dx}{x}, \tag{S10}$$

from which we get

$$\frac{y_0 + \Delta y}{y_0} = \frac{z_0 + \Delta z}{z_0} = \left(\frac{x_0 + \Delta x}{x_0}\right)^{-\nu_n}. \tag{S11}$$

Writing the deformation of the nucleus in the x-direction induced by the combination of pulling and pushing forces as Δx_{forces}, such that the new length of the nucleus is $x_{\text{forces}} = x_0 + \Delta x_{\text{forces}}$, we can then use Eq. (S11) to write the corresponding nuclear dimensions in the orthogonal directions as

$$y_{\text{forces}} = y_0 + \Delta y_{\text{forces}} = y_0 \left(1 + \frac{\Delta x_{\text{forces}}}{x_0}\right)^{-\nu_n} \tag{S12}$$

and

$$z_{\text{forces}} = z_0 + \Delta z_{\text{forces}} = z_0 \left(1 + \frac{\Delta x_{\text{forces}}}{x_0}\right)^{-\nu_n}, \tag{S13}$$

where $x_0/y_0/z_0$ denote the force-free width of the nucleus in the x /y /z-direction and ν_n is the Poisson ratio of the nucleus.

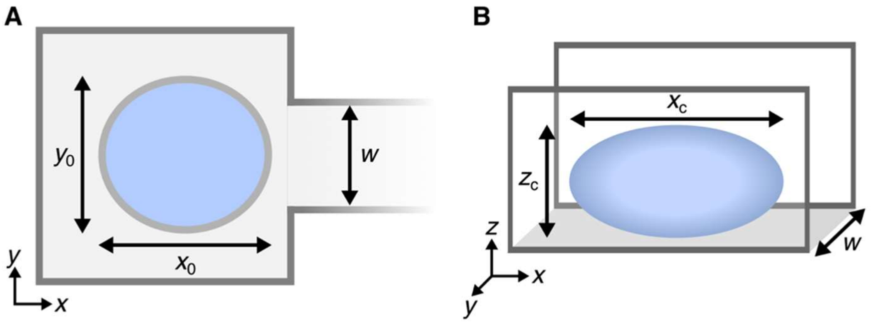

Fig. S8. Nuclear dimensions (A) on the island and (B) in the channel.

Now we add the confinement due to the channel of width w, such that the width of the nucleus in the channel $y_c = w$. Consequently, the diameter of the nucleus in the other dimensions is given by

$$x_c = x_{\text{forces}} \left(\frac{w}{y_{\text{forces}}}\right)^{-\nu_n} \tag{S14}$$

and

$$z_c = z_{\text{forces}} \left(\frac{w}{y_{\text{forces}}}\right)^{-\nu_n}. \tag{S15}$$

The aspect ratio between the two unconfined dimensions of the nucleus in the channel is then given by

$$AR_{\text{confined}} = \frac{x_c}{z_c} = \frac{x_0}{z_0}\left(\frac{x_{\text{forces}}}{x_0}\right)^{1+\nu_n}. \tag{S16}$$

If the pulling force is stronger than the pushing force, we expect that $\Delta x_{\text{forces}} > 0$ and thus $AR_{\text{confined}} > x_0/z_0 = AR_{\text{free}}$, while in the case that pushing forces are stronger than pulling forces, we expect $\Delta x_{\text{forces}} < 0$ and thus $AR_{\text{confined}} < AR_{\text{free}}$. We thus define the following shape parameter

$$\varepsilon = \frac{AR_{\text{confined}}}{AR_{\text{free}}} = \left(\frac{x_{\text{forces}}}{x_0}\right)^{1+\nu_n}. \tag{S17}$$

For $\varepsilon > 0$ pulling is the dominant force driving nucleus translocation, while for $\varepsilon < 0$ pushing dominates.

We do, however, not have direct experimental access to x^{forces}. To express ε in terms of measurable quantities, we use that $x_{\text{forces}} = x_c\left(\frac{w}{y_{\text{forces}}}\right)^{\nu_n}$ and $y_{\text{forces}} = y_0\left(\frac{x_{\text{forces}}}{x_0}\right)^{-\nu_n}$, such that

$$\frac{x_{\text{forces}}}{x_0} = \left[\frac{x_c}{x_0}\left(\frac{w}{y_0}\right)^{\nu_n}\right]^{\frac{1}{1-\nu_n^2}} \tag{S18}$$

and thus

$$\varepsilon = \left[\frac{x_c}{x_0}\left(\frac{w}{y_0}\right)^{\nu_n}\right]^{\frac{1+\nu_n^2}{1-\nu_n^2}}. \tag{S19}$$

2.3. Deducing the force balance on the nucleus

To relate the observed nuclear shapes to the difference of pulling and pushing forces, we consider the force balance acting on the nucleus as it moves through the cytosol (fig. S9). Since the Reynolds number of the nucleus is extremely low (*51*), its velocity is simply determined by this force balance. Since the drag force acts F_{drag} on the center of mass of the nucleus, we split it up with $1/2F_{\text{drag}}$ acting on either side of the nucleus, such that overall the nucleus experiences a drag force.

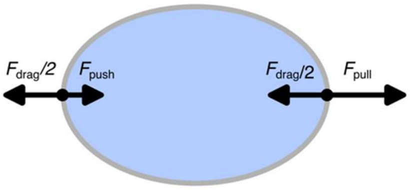

Fig. S9. Force balance on the nucleus. The drag force is split over the front and the half of the nucleus, such that the overall drag force is applied to the centre of the nucleus.

We then get the following net force acting on the front and the back of the nucleus:

$$F_{\text{front}} = F_{\text{pull}} - \frac{1}{2}F_{\text{drag}} \tag{S20}$$

and

$$F_{\text{back}} = F_{\text{push}} - \frac{1}{2}F_{\text{drag}}. \tag{S21}$$

At a constant velocity and shape of the nucleus, the forces at the front and the back must balance out and be equal to the elastic force F_{deform} generated by the nucleus due to its deformation:

$$F_{\text{deform}} = F_{\text{front}} = -F_{\text{back}} \tag{S22}$$

We can then rewrite $F_{\text{deform}} = (F_{\text{front}} - F_{\text{back}})/2$ in terms of the pulling and pushing forces as

$$F_{\text{deform}} = \frac{1}{2}\left(F_{\text{pull}} - F_{\text{push}}\right). \tag{S23}$$

As discussed above, the deformations of the nucleus can be approximated by the Hertz model, which yields the following expression of the deformation force in terms of the nuclear deformation along the direction of migration:

$$F_{\text{deform}} = \text{sign}\left(\Delta x^{\text{forces}}\right)\frac{4}{3}\frac{E_n}{1-\nu_n^2}\sqrt{\frac{x_0}{2}}\left|\frac{\Delta x^{\text{forces}}}{2}\right|^{3/2}. \tag{S24}$$

Together with Eq. (S18) the deformation force can then be written in terms of ε as

$$F_{\text{deform}} = \text{sign}\left(\varepsilon^{1/(1+\nu_n)} - 1\right)\frac{1}{3}\frac{E_n}{1-\nu_n^2}{x_0}^2\left|\varepsilon^{1/(1+\nu_n)} - 1\right|^{3/2}. \tag{S25}$$

This expression allows us to estimate the nuclear deformation forces from the experimentally observed nuclear shapes.

2.4. Plastic deformations and shape recovery

To analyze the viscoelastic response of nuclei getting deformed, we analyze the nuclear shapes depending on the channel width. We find that the shapes before and after the transition appear to be identical (fig. S10A) and that the nuclei recover their shape within minutes after the transition (fig. S10B). Unconfined nuclei appear to be slightly more elongated after transition through narrow channels (fig. S10B).

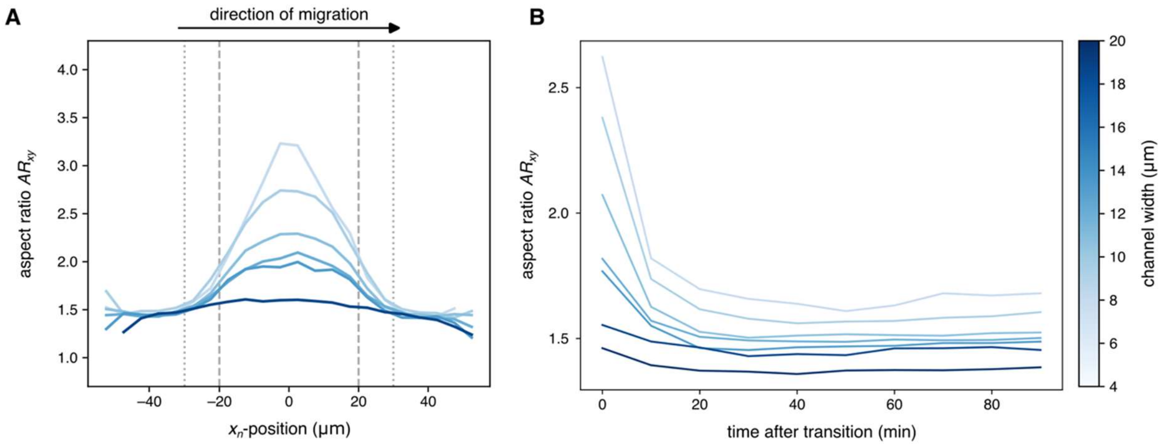

Fig. S10. Aspect ratio of the nucleus for different channel widths (x-dimension/y-dimension). (A) Aspect ratio with respect to the nuclear position. The cells are oriented such that they migrate from left to right. The grey dashed lines indicate the edges of the channel and the grey dotted lines the point at which the nucleus starts entering (left)/ fully left (right) the channel. **(B)** Nuclear shape recovery after a transition.

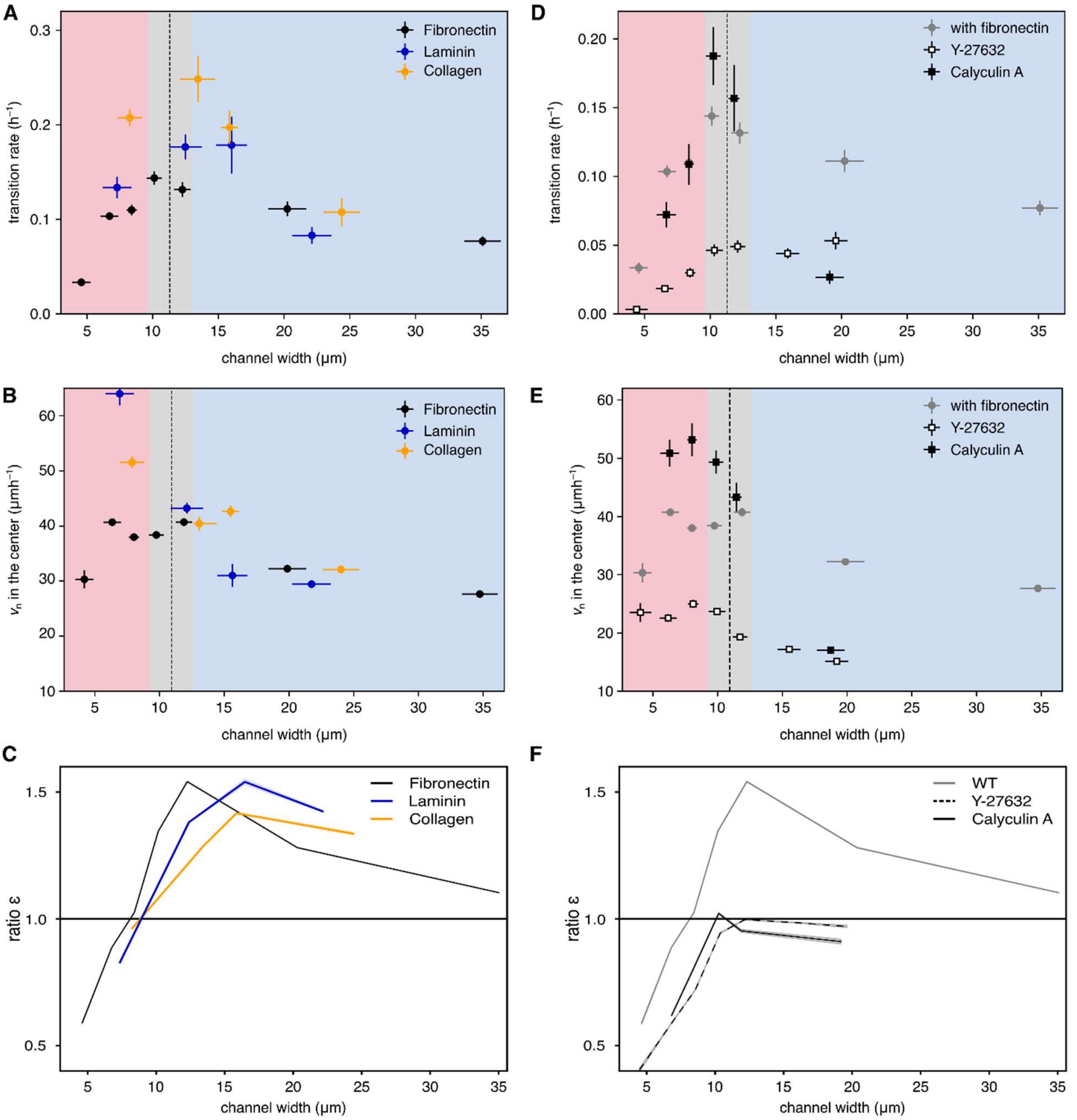

Fig. S11. Experimental data with different surface coatings and drug treatments. (A) - (C) Effect of different surface coatings. (A) Transition rates on laminin, collagen, and fibronectin coated patterns show a similar biphasic channel width dependence. Collagen and laminin result in overall slightly increased transition rates and a peak at a wider channel width compared to the case of fibronectin that is primarily discussed in the main text. **(B)** The nuclear velocities in the centre of the channel are largely independent of the surface coating at wider channel widths, but appear to be higher at lower channel width on patterns with laminin and collagen coating. **(C)** The biphasic channel width dependence of the nuclear shape parameter ε and its transition from $\varepsilon > 1$ to $\varepsilon < 1$ with decreasing channel width is also a generic feature of MDA-MB-231 cells irrespective of the surface coating. Similarly, to the transition rates, the maximum value of ε occurs at slightly wider channels than in the case of fibronectin coated patterns. **(D) - (F) Effect of increased (Calyculin A) and decreased (Y-27632) actomyosin contractility. (D)** Transition rates on Y-27632 and CalyculinA treated as well as wildtype MDA-MB-231 cells on fibronectin coated patterns. Under all conditions, cells show a similar biphasic channel width dependence. Y-27632 treatment results in

an overall decrease of transition rates. Calyculin A treatment results in an overall increase of transition rates compared to untreated cells. **(E)** Nuclear velocities at the channel centre behave similar to the transition rates. Y-27632 treated cells demonstrate a general decrease in nuclear velocities, while those treated with Calyculin A exhibited an overall increase.**(F)** Both treatments shift the relationship between pulling and pushing forces, favouring pushing forces compared to wildtype cells.

2.5. Cellular morphology depends on the predominant force generation mechanism

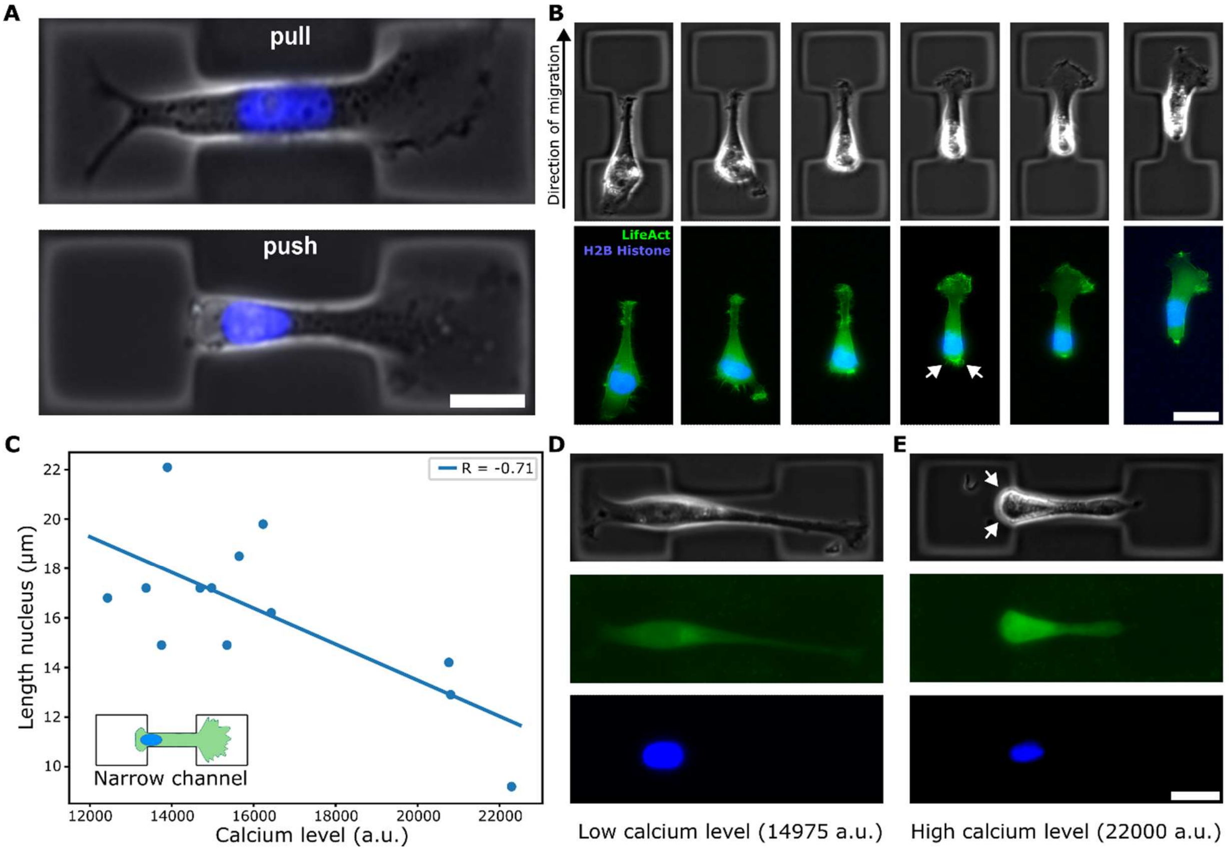

Fig. S12. Nuclear deformation forces depending on molecular composition of cells. (**A**) Pulling-based migration coincides with a more elongated nuclear shape in direction of migration while the rear of the cell still adheres to the previous chamber. Pushing-based migration coincides with a round shaped rear and compressed nucleus in the direction of migration (scale bar, 20 µm). (**B**) Representative image sequence of an MDA-MB-231 cell transmigrating the constriction, co-expressing fluorescently labelled histones (H2B-mcherry, blue) and F-actin (LifeAct-GFP, green). In pushing-based migration, the rear adopts a rounded shape, and the nucleus is compressed in the direction of migration, with filamentous actin enriched in the rear cell cortex upon nuclear entry in narrow constrictions (white arrows) (scale bar, 20 µm). (**C**) A negative correlation between nuclear length and observed calcium levels suggests that increased calcium levels have a more pronounced effect on pushing forces. (**D**) – (**E**) Exemplary images of MDA-MB-231 cells migrating through narrow channels, co-expressing fluorescently labelled histones (H2B-mCherry, blue). The utilization of the calcium indicator Calbryte520 AM reveals that increased calcium levels are associated with a rounded rear cell cortex (white arrows in **D**) (scale bar, 20 µm).

3.Inference of the dynamical signature of the confinement

To obtain information about the effect that physical confinement has on the effective dynamics of migrating cells, we employ an approach that was successfully used to infer dynamical models from trajectories of cells migrating on two dimensional micro-patterns (*11*, *35*) and apply it to our case of cells migrating in 3D confinement. At the core of this inference approach lies the observation that the dynamics of migrating cells, quantified through the position of the nucleus along the long axis of the pattern x_n, can be effectively described by an underdamped Langevin equation (*11*):

$$\ddot{x}_n = F(x_n, v_n) + \sigma\eta(t), \qquad \text{(S26)}$$

Where $F(x_n, v_n)$ is the deterministic (drift) contribution to the nuclear acceleration and $\eta(t)$ is a gaussian white-noise random variable that accounts for random fluctuations in the nuclear dynamics. The strength of these random fluctuations is given by σ.

In particularly, we are interested in how the deterministic contribution $F(x_n, v_n)$ changes with decreasing channel width. The chosen experimental procedure produces micro-cavities with a continuous range of different channel widths. To have enough trajectories to perform the inference procedure at different channel widths, we bin the data according to the measured width of the pattern in the centre of the channel. The resulting number of trajectories for every bin together with the average bridge widths are summarized in table S1.

To obtain a bias free estimate of the deterministic term $F(x_n, v_n)$, we employ a previously developed inference scheme, called Underdamped Langevin Inference(*38*). This method is based on an expansion of $F(x_n, v_n)$ in terms of manually chosen basis functions $\hat{c}_{\alpha\beta}(x_n, v_n)$. The order to which this expansion is performed determines the number of parameters that have to be estimated from the data. This means that this method can produce many different estimators for $F(x_n, v_n)$, which requires selection criteria that ensure that the chosen model is sufficiently precise and unique for each bridge width.

3.1. Symmetries of the basis functions

To reduce the number of free parameters as much as possible without constraining our self to a few leading order terms in the expansion of $F(x_n, v_n)$, we utilize the symmetries in our experimental setup to eliminate basis functions that do not match the systems symmetries. When choosing the centre of the pattern as the origin of our coordinate system, the symmetry of the pattern and the absence of any other anisotropic external cues implies that $F(-x_n, -v_n) = -F(x_n, v_n)$. To enforce this, we choose polynomial basis functions ${x_n}^l {v_n}^m$, where the combined order $l + m$ has to be odd.

3.2. Criteria for the model selection

Even with after the selection of a polynomial basis with the aforementioned symmetries, there is still freedom in the choice of the maximal included orders $l_{\max}$ and $m_{\max}$. Choosing $l_{\max}$ and $m_{\max}$ too low results in models of low complexity that might not be sufficient to capture the complex migration behavior. On the other hand, choosing $l_{\max}$ and $m_{\max}$ too high can lead to a number of model parameters that cannot be fully constrained from the data also resulting in a poor model. To select the right values for $l_{\max}$ and $m_{\max}$, we assess the predictive power of the inferred models.

The models are constrained by only using the instantaneous position, velocity and acceleration of the nucleus, without any knowledge about the dynamics on longer timescales. We use this to test our models by repeatedly simulating the inferred Langevin equation with random initial conditions to generate a synthetic dataset containing the same number of cells as the experimental dataset. When cells exit the experimentally sampled region in phase space we terminate the simulation for that cell. We then calculate a number of key statistics that describe the long timescale behaviour of the population of cells. These include the probability distribution of the nuclear position $p(x_n)$ and velocity $p(v_n)$ as well as the dwell time distribution $p(\tau)$.

To denoise the distributions, we estimate $p(x_n)$ and $p(v_n)$ in parameter-free way using the kernel-density estimate with Gaussian kernels implemented with the python library scipy (*52*), which closely matches the distributions obtained from histograms (fig. S9, A and B). To estimate the dwell time distribution $p(\tau)$ one needs to account for the finiteness of the trajectories. We did this by using the python library liveliness (*53*). We found that a generalized gamma model was in close agreement with the parameter-free Kaplan-Maier estimator but was numerically more well behaved (fig. S13C), such that we chose this model for the calculation of $p(\tau)$.

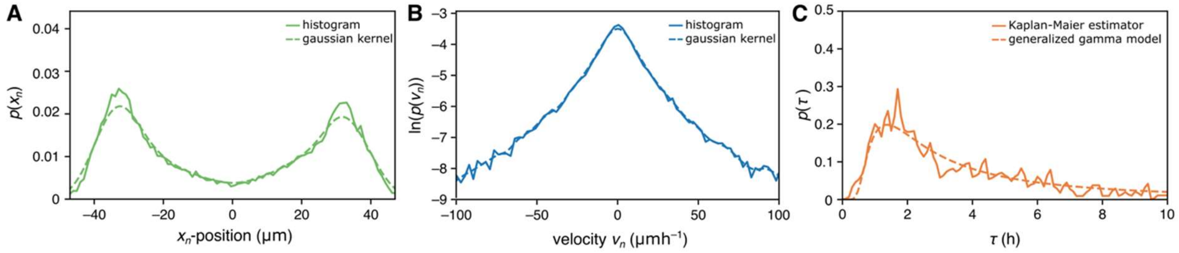

Fig. S13. Denoising of the experimental statistics used for model selection. Considered were the probability distributions of (A) the nuclear velocities (B) the nuclear position (C) the dwell times.

The fact that all three of those quantities are probability distributions allows for a simple and comparable quantification of the difference between the experimental and the synthetic dataset. Due to its numerical stability and its boundedness, we choose to quantify the difference between the distributions obtained from the experiment and the simulations with the so-called Hellinger distance $H(p_{\text{exp}}, p_{\text{inf}})$ (*54*), which takes values between 0 (perfect agreement between p_{exp} and p_{inf}) and 1 (complete disagreement between p_{exp} and p_{inf}). To quantify the accuracy of the inferred models, we sweep over $l_{\max}$ and $m_{\max}$ and infer models from one half of the available data (fig. S14). We then simulate the model and calculate $p(x_n)$, $p(v_n)$ and $p(\tau)$ from this synthetic data set. We do the same for the half of the data that was not used for training and compute $H(p_{\text{exp}}, p_{\text{inf}})$ for all three statistics. As shown in fig. S10, there are strong differences in the performance of the different models.

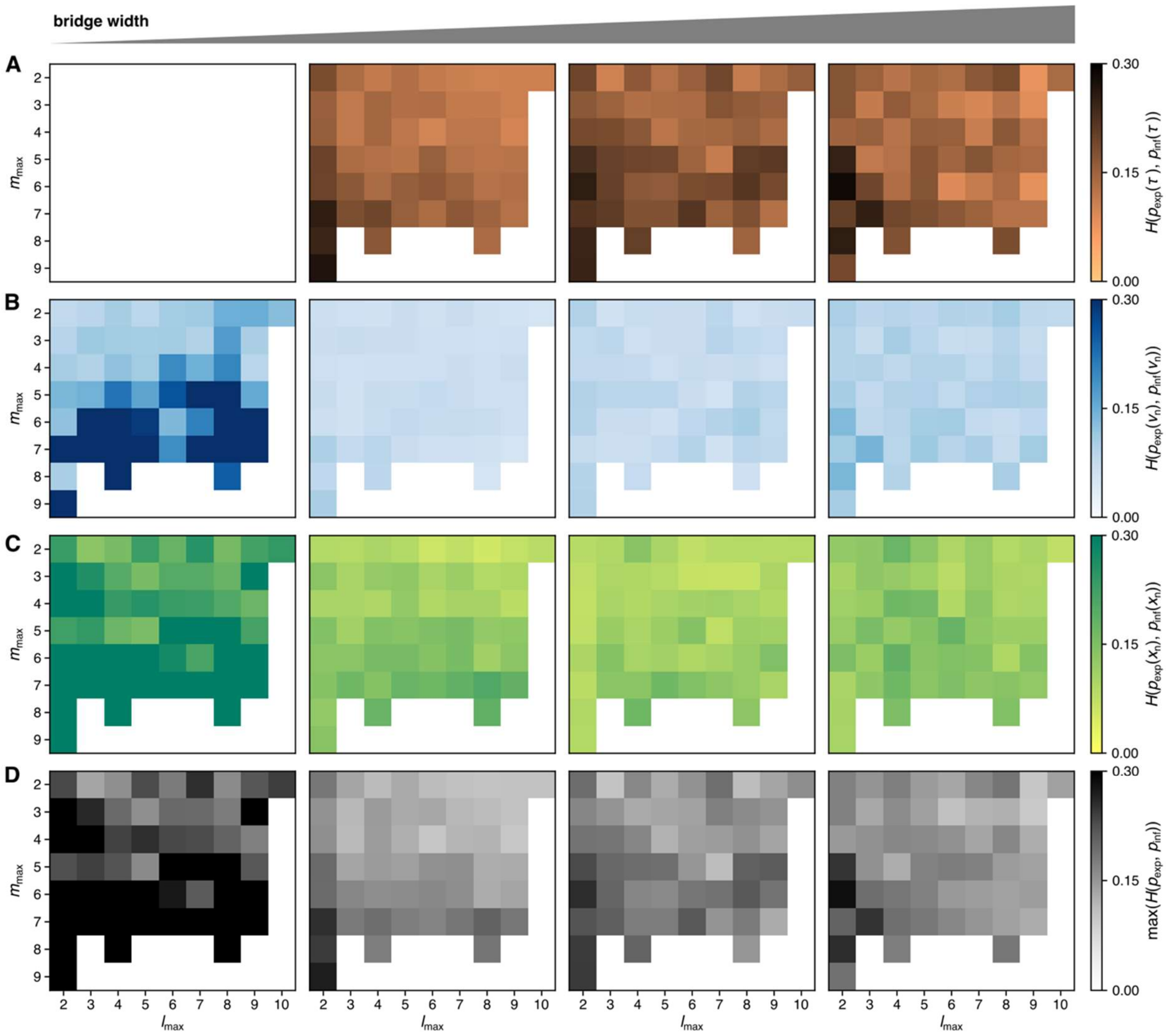

Fig. S14. Overview of the Hellinger distances between experimental and model statistics for models of different orders. (A) Hellinger distances of the dwell time distributions. **(B)** Hellinger distances of the velocity distributions. **(C)** Hellinger distances of the position distributions. **(D)** Maximal Hellinger distance of the three considered statistics.

3.3. Uniqueness of the inferred models

To choose a value for H_{thresh}, we started out with a value of $H_{\text{thresh}} = 0.15$, which ensures that already large parts of the probability distributions are reproduced by the model. A model was accepted if all three probability distributions scored a value of $H(p_{\text{exp}}, p_{\text{inf}}) < H_{\text{thresh}}$. We found however that this criterium was not strict enough to lead to unique models, but instead found large differences between the different models. We then gradually decreased the threshold until we obtained a selection of quantitively identical models for each bridge width. This was the case for a value of $H_{\text{thresh}} = 0.13$ (see fig. S15 for $H_{\text{thresh}} = 0.14$ and fig. S16 for $H_{\text{thresh}} = 0.13$).

For the narrowest bridge widths ($w \approx 5$ µm) almost no transitions were observed anymore. As a consequence, the dwell time distribution could not be properly estimated and was excluded from as a selection criterium for this bridge width. Further, the model was not able to reproduce the sharply peaked positional probability distributions well enough to pass the threshold. Since we are however

more interested in the dynamical properties of our model, we loosened the selection criterium in that case to $H_{thres} = 0.14$. The models that were shown in the main text are given in table S2.

Bridge width	4 µm	7 µm	9 µm	12 µm
$l_{\max}$	3	9	8	7
$m_{\max}$	2	3	2	3

Table S2. Orders of the basis functions used to infer the models discussed in the main text.

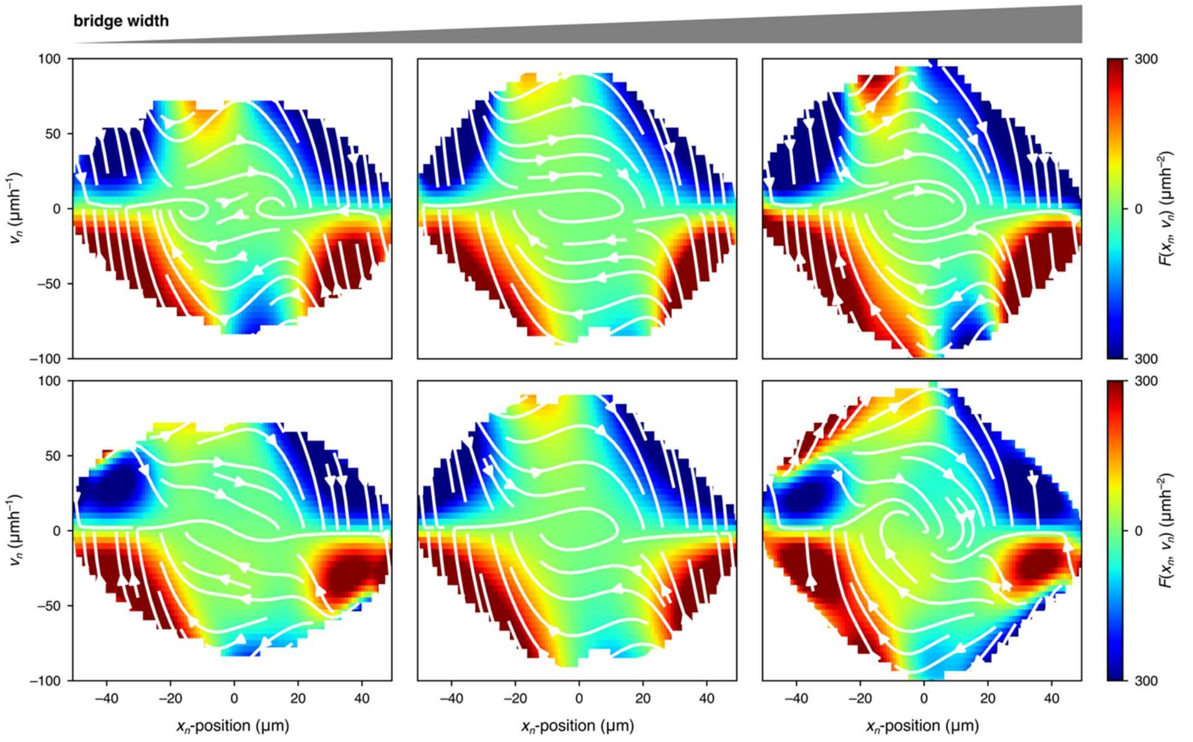

Fig. S15. Comparison between exemplary models with a threshold of 0.14. Shown are the bridge widths 7 µm, 9 µm and 12 µm.

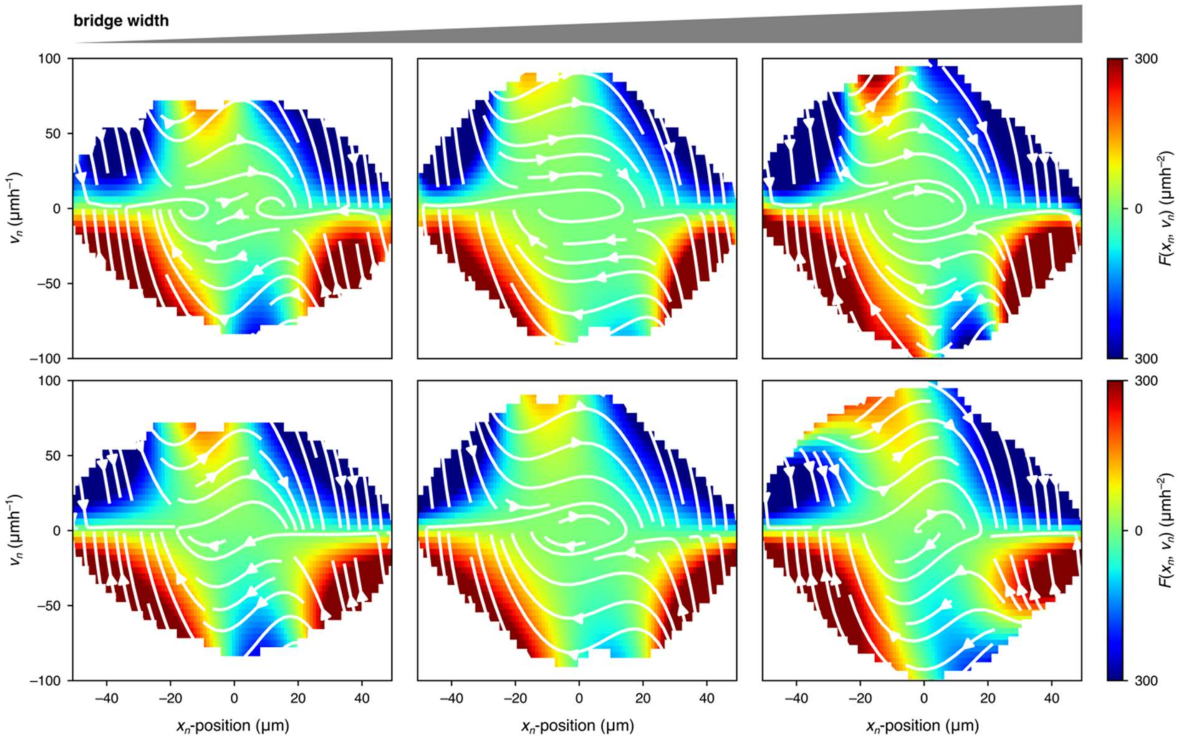

Fig. S16. Comparison between exemplary models with a threshold of 0.13. Shown are the bridge widths 7 µm, 9 µm and 12 µm.

4.Mechanistic model

To model the effect of 3D confinement on mesenchymal cell migration, we adapt a model for cell migration on 2D dumbbell-shaped micro-patterns that we previously constrained from experimental data (*35*).

4.1. General structure of the model

The model accounts for three central degrees of freedom of the cell: the nuclear position, the position of the leading protrusion of the cell and the polarity of the cell that drives protrusion growth (fig. 6B in the main text). The coupling between nucleus and protrusion is modelled by linear elastic spring connecting the two. The dynamics of the nucleus are then given by the following equation of motion:

$$\gamma(x_n)\dot{x}_n = k_n(x_p - x_n) + f_{\text{push}}(x_n) + f_{\text{channel}}(x_n) \tag{S27}$$

Here, $\gamma(x_n)$ accounts for spatial variations in the nuclear friction coefficient due to reduced amount of adhesion bonds in the channel compared to the islands, k_n denotes the effective spring constant that acts on the nucleus, $f_{\text{push}}(x_n)$ is the pushing force generated by rear contractions of the cell and $f_{\text{channel}}(x_n)$ denotes the force due to the nuclear deformations induced by the channel.

The dynamics of the protrusion coordinate are given by

$$\dot{x}_p = -k_p(x_p - x_n) - \partial_{x_p}V(x_p) + rP(t), \tag{S28}$$

where k_p is the effective spring constant that acts on the nucleus and the parameter r converts the cell polarity into a protrusion growth rate. The protrusion formation is limited to the region of the dumbbell-shaped micro-pattern. To account for this, a repulsive potential $V(x_p) = (x_p/x_{\text{boundary}})^8$ is introduced that acts on the protrusion coordinate. Additionally, to account for the internal organization of the cell, a polarization force $P(t)$ acts on the protrusion coordinate driving protrusion formation in the direction of polarization.

In previous work, we found that the polarization force is sensitive to the geometry of the pattern (*35*): In the absence of confinement, the cell is on average unpolarized and $P(t)$ fluctuates around zero. In contrast, in the case of narrow confinement of the protrusion, the cell polarization gets reinforced and $P(t)$ takes a finite value. This behavior is summarized in the following equation:

$$\dot{P} = -\alpha(x_p)P - \beta P^3 + \sigma\xi(t) \tag{S29}$$

Here, the geometry sensitive parameter determines the value of the stable fixed point P^* of $P(t)$ with $P^* = \pm\sqrt{-\alpha/\beta}$ for $\alpha < 0$ and $P^* = 0$ for $\alpha \geq 0$. The constant $\beta > 0$ ensures that $P(t)$ remains bounded at all times. The term $\sigma\xi(t)$ denotes a Gaussian white noise-process of strength σ and with $\langle\xi(t)\rangle = 0$ and $\langle\xi(t)\xi(t')\rangle = \delta(t - t')$ that accounts for stochastic fluctuations in the internal organization of the cell.

To account for the increased confinement in the centre of the pattern, we gradually decrease α towards the center of the pattern according to

$$\alpha(x_p) = \begin{cases} \alpha^{\min} + \frac{\alpha_0 - \alpha^{\min}}{2}\left(1 - \cos\left(\frac{x_p \pi}{L_\alpha}\right)\right), |x_p| < L_\alpha \\ \alpha_0, \qquad \text{else} \end{cases}. \tag{S30}$$

The parameter $\alpha^{\min}$ decreases with decreasing bridge width according to (*41*)

$$\alpha^{\min} = -\alpha_1 + \alpha_2 w. \tag{S31}$$

For channel widths beyond the unconfined width of the cell w_{free}, we expect the channel width to only have a minimal effect on the experiment, such that in that regime, we assume that $\alpha^{\min} = \alpha_0$.

4.2. Nuclear friction

In the absence of nuclear deformations, the nuclear friction was found to be reduced in the centre of dumbbell shaped patterns (*35*). A possible explanation for this is the reduced number of focal adhesions due to the reduced adhesive area in the constriction (*41*). In the presence of physical walls that strongly confine the nucleus, however, we expect that the nuclear friction increases in the channel due to interactions with the walls. To account for the varying friction that the nucleus experiences on the pattern, we gradually adapt $\gamma(x_n)$ from its value of 1 on the island towards a value γ^{centre} in the centre of the pattern. For this we follow the approach used in (*35*) and use the following analytic expression for $\gamma(x_n)$:

$$\gamma(x_n) = \gamma^{\text{centre}} + \frac{1 - \gamma^{\text{centre}}}{2}\left(1 - \cos\left(\frac{x_n \pi}{L_{\text{sys}}}\right)\right) \tag{S32}$$

Here, $2L_{\text{sys}}$ denotes the total length of the pattern. If the cell is completely unconfined ($w \geq w_{\text{free}}$), we simply expect $\gamma^{\text{centre}} = 1$.

When the cell is confined but the nucleus is not deformed ($2R_n \leq w < w_{\text{free}}$), we expect a linear decrease of γ^{centre} with channel width due to the reduced adhesive area compared to the unconfined case (*35*, *41*). Finally, in the presence of nuclear deformations ($w < 2R_n$)), we expect a non-linear increase of the nuclear drag with reducing channel width (*55*). Taken together, we use the following expression for the nuclear friction in the channel:

$$\gamma^{\text{centre}} = \begin{cases} 1, w > w_{\text{free}} \\ \frac{1 + \gamma_1 w}{1 + \gamma_1 w_{\text{free}}}, 2R_n < w \leq w_{\text{free}} \\ \frac{1 + 2\gamma_1 R_n}{1 + \gamma_1 w_{\text{free}}}\left(1 + \frac{\gamma_2}{w}\right), w \leq 2R_n \end{cases} \tag{S33}$$

4.3. Deformation barrier

Motivated by the observation that the 3D channel appears as an effective barrier in the inferred dynamics of the nucleus for channel widths below the size of the nucleus (see fig. 3 in the main text) together with the steep decrease in transition rates in that regime, we account for nuclear deformations induced by the channel through a potential barrier, such that

$$f_{\text{channel}}(x_n) = -\partial_{x_n} W(x_n). \tag{S34}$$

As the nucleus moves into the confinement, it is compressed in the orthogonal direction, which leads to a lengthening of the nucleus. The confinement exerts a force opposite to the direction of movement onto the nucleus until it has completely entered the bridge. We thus use the following expression for the potential barrier:

$$W(x) = W_{\max} \begin{cases} \frac{x}{L_W} - \pi^{-1} \cos\left(\frac{\pi x}{L_W}\right) \sin\left(\frac{\pi x}{L_W}\right), 0 < x < L_W \\ 0, \quad \text{else} \end{cases}, \tag{S35}$$

where $L_W = L_b/2 + R_n$ with R_n being the average radius of the unconfined nucleus. Since the pattern and consequently also the potential is symmetric, we can calculate the value of the potential barrier at position x_n through $W(x = L_W - |x_n|)$. We thus get the channel-induced deformation force

$$f_{\text{channel}}(x_n) = -\partial_x W(x) \partial_{x_n} x|_{x=L_W-|x_n|} = \text{sign}(x_n) \partial_x W(x)|_{x=L_W-|x_n|}. \tag{S36}$$

The observed 3/2-scaling of the nuclear normal forces with the nuclear deformations represents a lower bound for the dependence of the force opposing the nuclear deformations. We thus used the following expression for $W_{\max}$, which yielded good agreement between our model and the experiments:

$$W_{\max} = W_0 (2R_n - w)^{3/2} \tag{S37}$$

4.4. Pushing force

There is recent evidence that in confinement, cells can generate pushing forces to move the nucleus through a constriction (*32*, *33*). In the absence of confinement, we assume that the pushing force can be neglected due to the unhindered fluid exchange between the front and the rear of the cell. To account for the gradual increase in nuclear deformation, we apply an increasing pushing force that acts directly onto the nucleus as the nucleus moves into the centre of the channel. The pushing force is then given by

$$f_{\text{push}}(x_n) = \text{sign}(x_p - x_n) f_{\max}(w) \cos\left(\frac{\pi x_n}{L_b}\right). \tag{S38}$$

To find an expression for $f_{\max}(w)$, we consider the case of a nucleus that is completely confined to a width w in the y-direction. These pushing forces are thought to be triggered by strong nuclear deformations that lead to Ca^{2+} release and consequently increased myosin recruitment to the cortex (*19*, *20*). This leads to increased cortical tension and thus an increased pushing force due to the increased Laplace pressure The pushing force in confinement is then proportional to the increase in cortical tension $\Delta\tau_{\text{cortex}}$ and the projected area S of the nucleus in the plane orthogonal to the direction of migration (*30*)

$$f_{\max}(w) \propto S\big(w(x_n)\big) \Delta\tau_{cortex}. \tag{S39}$$

Since we expect the dominant contribution to the bridge width dependence of $S(w(x_n))$ to be the compression imposed by the side walls of the channel, we can estimate the projected area of the nucleus as

$$S(w(x_n)) = S_0 \left(\frac{w(x_n)}{2R_n}\right)^{1-\nu_n}, \tag{S40}$$

where S_0 is the projected area of the undeformed nucleus.

The calcium release upon physical confinement of the nucleus is associated with stretch-sensitive calcium channels in the perinuclear endoplasmic reticulum (*19*). To relate the change in Ca^{2+} concentration in the cytosol to the degree of confinement, we assume that the concentration is proportional to the probability that a channel is open. We use a simple mechanical model for the opening probability of the calcium channels, which gives us (*56*)

$$n^{Ca^{2+}} \propto \frac{1}{1 + \exp\left((\Delta E - \Delta A \tau_{ER}(w(x_n))/k_B T\right)}, \tag{S41}$$

where ΔE is the energy difference between the opened and closed state of the calcium channel and ΔA is the change in area of the channel upon opening, caused by the tension in the perinuclear endoplasmic reticulum τ_{ER}.

We assume a linear scaling of τ_{ER} with the nuclear deformation $2R_n - w(x_n)$ to leading order. In the range of nuclear deformations probed in our experiments, we expect the exponential part of the sigmoidal function in the previous equation dominates. This assumption is supported by measurements of the cortical myosin concentration under confinement in primary progenitor stem cells cultured from zebrafish embryos (*20*), which probed confinement heights down to 7 μm without observing a clear deviation from an exponential dependence. We can then write in Ca^{2+} concentration as

$$\Delta n^{Ca^{2+}} = n_0^{Ca^{2+}} \left(e^{\frac{2R_n - w(x_n)}{h^*}} - 1\right), \tag{S42}$$

where h^* is the characteristic confinement height of calcium release.

In principle the cortical tension could show a non-linear response on the calcium concentration due to effects like contraction induced Rho release (*33*) or saturation of the contractility. However, to keep our model simple, we only consider a linear dependence of the cortex tension on the calcium concentration. We checked that allowing for higher order terms does not change our results qualitatively. We can then express the pushing force explicitly as a function of the confinement width of the nucleus $w(x_n)$ as

$$\begin{aligned} f_{max}(w) &= S_0 \tau^0_{\text{cortex}} \left(\frac{w(x_n)}{2R_n}\right)^{1-\nu_n} \left(e^{\frac{2R_n - w(x_n)}{h^*}} - 1\right) \\ &\approx S_0 \tau^0_{\text{cortex}} \left(\frac{w(x_n)}{2R_n}\right)^{1-\nu_n} \frac{2R_n - w(x_n)}{h^*} \end{aligned} \tag{S43}$$

where τ^0_{cortex} denotes the cortical tension in the absence of nuclear deformations.

4.5. Implementation and simulations

The set of closed Eqs. (27) – (29) was implemented in Python and numerically solved with a standard forward Euler scheme. To ensure comparability to the experimental data, we sampled values every 10 min for the simulated trajectories and chose the trajectory length such that it corresponded to 50 h, which is similar to the maximum observation time in the experiment.

Whenever possible the values of the model parameters were taken previous work (*35*, *41*) or set to match the experimental geometry. The remaining parameters used in the simulations were constrained by fitting the model to a number of key experimental statistics (nuclear deformation forces, transition rates and nuclear velocities). The resulting parameter values are given in table S3.

Parameter	value	parameter	value
L_{sys} (μm)	55 (experiment)	R_n (μm)	6 (experiment)
L_c (μm)	40 (experiment)	k_n (h^{-1})	1.2 (fit)
x_{boundary} (μm)	$0.4 \times L_{sys}$ (*35*)	k_p (h^{-1})	2.4 (fit)
σ ($\mu\mathrm{mh}^{-3/2}$)	100 (*35*)	L_α (μm)	25 (fit)
α_0 (h^{-1})	1.1 (fit)	W_0 ($\mu\mathrm{m}^{1/2}\ \mathrm{h}^{-1}$)	40 (fit)
α_1 (h^{-1})	2.1 (fit)	$\frac{S_0 \tau^0_{cortex}}{h^*}$ ($\mu\mathrm{m\ h}^{-1}$)	16 (fit)
α_2 (h^{-1})	0.005 (fit)	r	2 (fit)
β (h^{-1})	0.0001 (*35*)	γ_1 ($\mu\mathrm{m}^{-1}$)	0.3 (*41*)
w_{free} (μm)	25 (experiment)	γ_2 ($\mu\mathrm{m}^{-1}$)	15 (fit)

Table S3. Model parameters.

4.6. Calculation of the effective underdamped dynamics

To compare the simulated dynamics with the inferred dynamics, we need to find a formulation of the model in terms of an underdamped Langevin equation (Eq. (1) in the main text). To derive an expression of the deterministic term $F(x_n, v_n)$ from our mechanistic model, we follow previous work (*35*) and rewrite the equations for the nuclear and the protrusion coordinate as

$$\gamma(x_n)\dot{x}_n = k_n(x_p - x_n) + f_{\mathrm{push}}(x_n) + f_{\mathrm{channel}}(x_n) \equiv f_n(x_n, x_p) \tag{S44}$$

and

$$\dot{x}_p = -k_p(x_p - x_n) - \partial_{x_p} V(x_p) + rP(t)) \equiv f_p(x_n, x_p) + rP(t). \tag{S45}$$

This allows us to calculate $F(x_n, v_n)$ according to Eq. (1) from the main text as

$$F(x_n, v_n) = \langle \dot{v}_n | x_n, v_n \rangle \overset{\text{Eq. (S44)}}{=} \left\langle \frac{df_n(x_n, x_p)}{dt} \middle| x_n, v_n \right\rangle = \left\langle \frac{df_n}{dx_n} v_n + \frac{df_n}{dx_p} \dot{x}_p \middle| x_n, v_n \right\rangle$$

$$\overset{\text{Eq. (S45)}}{=} v_n \frac{df_n}{dx_n} + \frac{df_n}{dx_p} \langle f_p(x_n, x_p) + rP(t) | x_n, v_n \rangle \qquad \text{(S46)}$$

$$\overset{\text{Eq. (S44)}}{=} v_n \frac{df_n}{dx_n} + \frac{df_n}{dx_p} f_p(x_n, x_p)\Big|_{x_p = x_n + k_n^{-1}(\gamma(x_n) v_n - f_{\text{push}}(x_n) - f_{\text{channel}}(x_n))} + \frac{df_n}{dx_p} \langle rP(t) | x_n, v_n \rangle$$

While most terms in Eq. (S46) can be calculated analytically, $\langle rP(t) | x_n, v_n \rangle$ needs to be determined numerically. The resulting $F(x_n, v_n)$ and corresponding confinement signature maps are shown in fig. S17C and D.

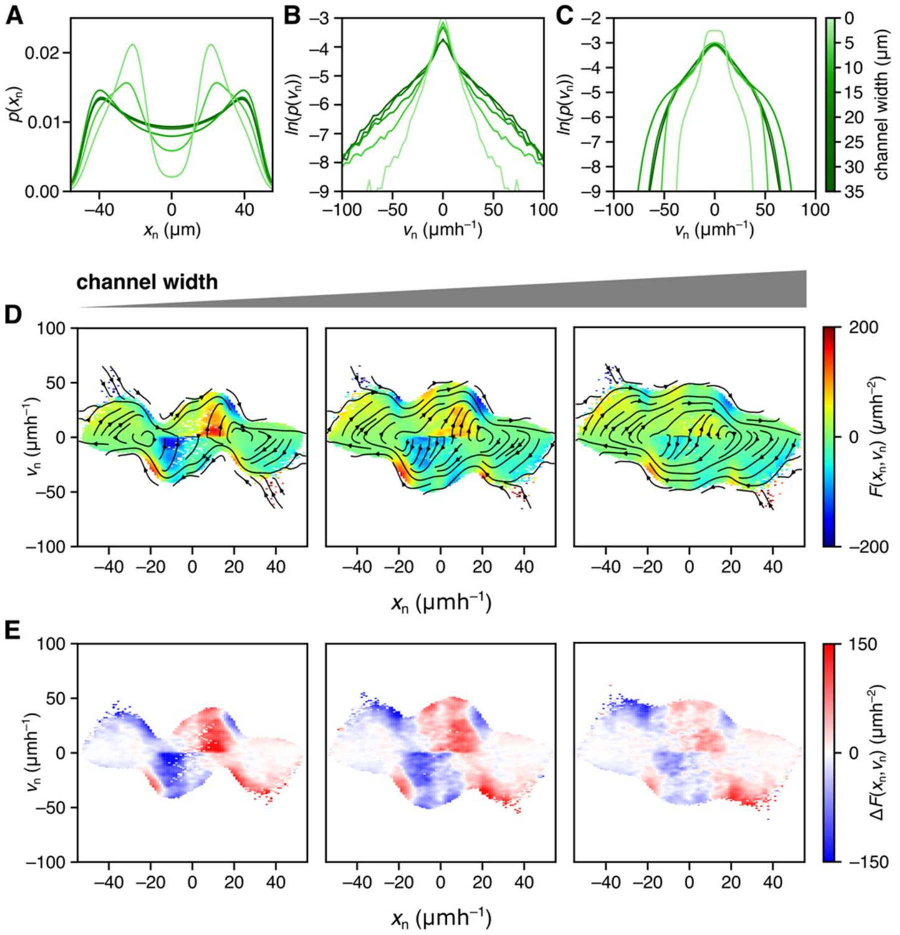

Fig. S17. Further comparison between simulation and experiment. (A) Probability distribution of the nuclear position at varying channel widths predicted from the mechanistic model. **(B), (C)** Probability distributions of the nuclear velocities at varying channel widths obtained experimentally (B) and via simulations (C). **(D), (E)** Effective underdamped dynamics of the model. Deterministic contribution (D) and corresponding confinement signature maps of the effective underdamped dynamics of the model (from left to right: 4 µm, 7 µm, 9 µm).

4.7. Comparison of experimental data and model predictions

We find that both the distribution of the nuclear position and velocity show semi-quantitative agreement with the experimental data. In particular, the model captures the overall dependence of the distributions on the channel width, with the appearance of clear peaks of the x_n-distribution in the chambers and pronounced minima in the channel at narrow channel widths and an overall narrowing of the nuclear velocities with increasing confinement (fig. S17A-C). We find that the location of the peaks in the x_n-distribution deviates between experiment and simulations. For the simulation we find a maximum near the entrance of the channel, while in the experiments the maximum is located more towards the centre of the chamber. In line with this, we find that the biggest differences between the inferred dynamics and the effective underdamped dynamics of the model are found on the islands, while our model captures the dynamics of the actual transition accurately (main text fig. 3 and fig. S17D and E). From assessment of the bright-field videos, we found that after a failed transition attempt during which the nucleus is placed near the entrance of the channel, the cells often retract their nucleus back to the centre of the channel for an extended period of time before it starts a new attempt. Since our model is designed to capture the interactions between the confinement and the cell and not necessarily the cellular behaviour in the chamber, we did not include a mechanism that could account for this in our model in order to keep our mechanistic model as simple as possible while still capturing the essence of the cell confinement interactions.

Beyond the differences between the model and the experiments in the chambers, we also find that the experimental velocity distribution fall of slower (fig. S17, B and C). This does however mostly affect the region of extremely high velocities (>50 μm h^{-1}), which occur extremely rare, such that they have little effect on the population averaged migration dynamics.

The model is also able to capture more detailed aspects of the cellular transition dynamics (fig. S18). In particular the strong channel width dependence of the reentry time between consecutive transitions and to the relatively low channel width dependence of the time spent in the channel during a transition are captured correctly (fig. S18A). Further, the model qualitatively captures the biphasic channel width dependence of the success rate of transition events (fig. 17B). At narrow channel widths, there is a quantitative difference between the model prediction and the experimental observation. This is likely to be another consequence of the fact that our model does not capture the fact that after failed attempts cells often retract their nucleus back to the centre of the channel for an extended period of time at narrow channel widths. Thus at the same overall transition rate our model will predict a much lower transition success rate than observed experimentally.

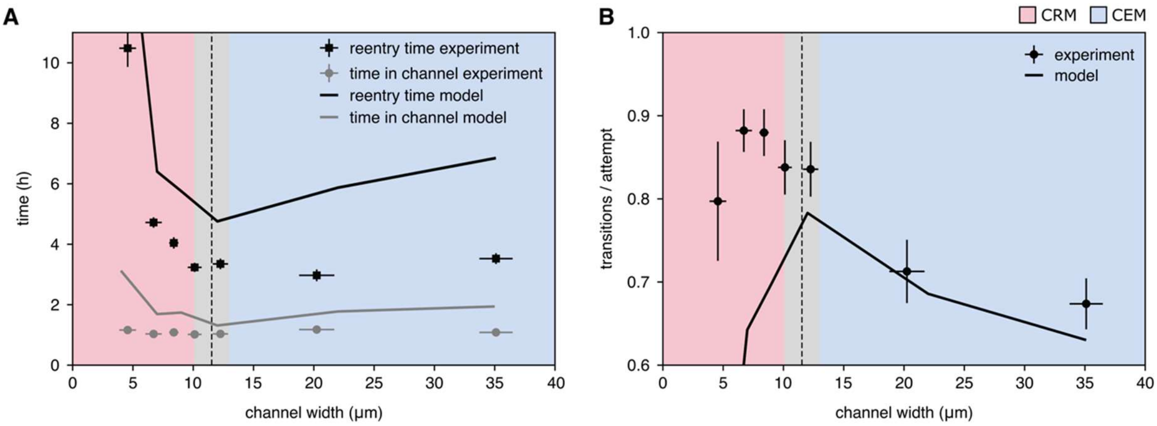

Fig. S18. Transition Statistics (A) The average time between transitions can be decomposed into the time that cells spend on the island (black) and in the channel (grey). The time in the channel is

defined as the time that the center of mass of the nucleus is inside the channel during a successful transition. The re-entry time is then defined as the total time between consecutive transitions minus the time spent in the channel during the successful transition. While the time in the channel only shows a weak channel width dependence, the time spend on the island strongly increases with decreasing channel width in the confinement reduced migration regime (CRM). These trends are also reproduced by our mechanistic model (lines). **(B)** The fraction of successful transitions shows a biphasic behaviour with a peak around 7 μm that is also reproduced by the mechanistic model.

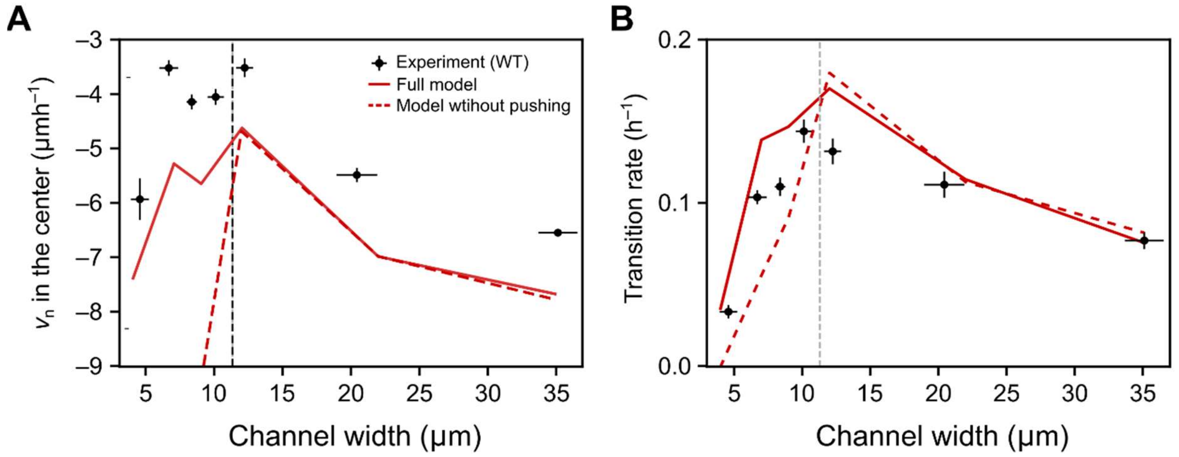

Fig. S19. The role of cellular pushing forces for the transmigration dynamics. Comparison between **(A)** the transition rates and **(B)** the nuclear velocities predicted full model (solid red line, parameters given in Table S3), a model with the same parameters but the lack of pushing forces (dashed red line, $S_0\ \tau^0_{\mathrm{cortex}}/h^* = 0$), and measured experimentally (black data points, same data as in figure 6D, E). While transition rates are only mildly affected by the lack of pushing forces, the model without cellular pushing forces fails to capture the nuclear velocities for channel widths below the nuclear width (indicated by the vertical dashed line).

5. Other Supplementary Materials descriptions

Movie S1.
Single MDA-MB-231 cell transitioning repeatedly between the square islands of the dumbbell-shaped cavities. Transitions are usually preceded by the formation of a protrusion along the bridge. The cells are co-expressing histone-2B mCherry and F-actin (LifeAct-TagGFP2) to allow the visualization of the nucleus and actin distribution. The bridge width shown in movie 1 is W = 16 μm. Scale bar: 20 μm.

Movies S2 and S3.
Single MDA-MB-231 cells transitioning repeatedly between the square islands of the dumbbell-shaped cavities. Transitions are usually preceded by the formation of a protrusion along the bridge. The cells are co-expressing histone-2B mCherry to allow automated tracking of cell positions. The bridge widths shown in movies 2 and 3 are W = 18 and 5 μm respectively. Scale bar: 20 μm.

Movie S4.
4D experiment of a single MDA-MB-231 cell transitioning through the thin bridge. Cell nucleus is shown in blue and embedded fluorescent-nanobeads are shown in red. The cell is co-expressing histone-2B mCherry, enabling the determination of the nuclear volume at each time point. Scale bar: 20 μm.

Movie S5.
Single MDA-MB-231 cell transitioning through the thin bridge. Cell nucleus and embedded fluorescent-nanobeads are shown in grey. The hydrogel composition was chosen soft to allow recognizable bead displacement due to the nucleus squeezing through the constriction. The length and colour of the arrows represent the amount of displacement from their neutral position and the direction in which the arrow shows indicate the direction in which the deformation went from its neutral position. The bridge width shown in movie 5 is W = 6 μm. Scale bar: 20 μm.

Movie S6.
Single MDA-MB-231 cell transitioning repeatedly between the square islands of the dumbbell-shaped cavities without fibronectin coating. Transitions are usually preceded by the formation of a protrusion along the bridge. The cell is co-expressing histone-2B mCherry to allow automated tracking of the cell position. The bridge width shown in movie 6 is W = 9 μm. Scale bar: 20 μm.

Movie S7.
Single MDA-MB-231 cell treated with the ROCK-inhibitor Y27632 transitioning repeatedly between the square islands of the dumbbell-shaped cavities. Transitions are usually preceded by the formation of a protrusion along the bridge. The cell is co-expressing histone-2B mCherry to allow automated tracking of the cell position. The bridge width shown in movie 7 is W = 9 μm. Scale bar: 20 μm.

Movie S8.
Single MDA-MB-231 cell treated with the myosin activator Calyculin A transitioning repeatedly between the square islands of the dumbbell-shaped cavities. Transitions are usually preceded by the formation of a protrusion along the bridge. The cell is co-expressing histone-2B mCherry to allow automated tracking of the cell position. The bridge width shown in movie 8 is W = 9 μm. Scale bar: 20 μm.

Table S4.
Primer and pDNA sequences used to transfect MDA-MB-231 cells with both EGFP-Paxillin and EGFP-DN-KASH.

REFERENCES AND NOTES

1. S. Barrientos, O. Stojadinovic, M. S. Golinko, H. Brem, M. Tomic-Canic, PERSPECTIVE ARTICLE: Growth factors and cytokines in wound healing. *Wound Repair Regen.* **16**, 585–601 (2008).

2. A. D. Doyle, R. J. Petrie, M. L. Kutys, K. M. Yamada, Dimensions in cell migration. *Curr. Opin. Cell Biol.* **25**, 642–649 (2013).

3. F. Qu, F. Guilak, R. L. Mauck, Cell migration: Implications for repair and regeneration in joint disease. *Nat. Rev. Rheumatol.* **15**, 167–179 (2019).

4. K. M. Yamada, M. Sixt, Mechanisms of 3D cell migration. *Nat. Rev. Mol. Cell Biol.* **20**, 738–752 (2019).

5. C. D. Paul, P. Mistriotis, K. Konstantopoulos, Cancer cell motility: Lessons from migration in confined spaces. *Nat. Rev. Cancer* **17**, 131–140 (2016).

6. S. B. Khatau, R. J. Bloom, S. Bajpai, D. Razafsky, S. Zang, A. Giri, P.-H. Wu, J. Marchand, A. Celedon, C. M. Hale, S. X. Sun, D. Hodzic, D. Wirtz, The distinct roles of the nucleus and nucleus-cytoskeleton connections in three-dimensional cell migration. *Sci. Rep.* **2**, 488 (2012).

7. P. Friedl, K. Wolf, J. Lammerding, Nuclear mechanics during cell migration. *Curr. Opin. Cell Biol.* **23**, 55–64 (2011).

8. C. M. Denais, R. M. Gilbert, P. Isermann, A. L. McGregor, M. te Lindert, B. Weigelin, P. M. Davidson, P. Friedl, K. Wolf, J. Lammerding, Nuclear envelope rupture and repair during cancer cell migration. *Science* **352**, 353–358 (2016).

9. A. C. Rowat, D. E. Jaalouk, M. Zwerger, W. L. Ung, I. A. Eydelnant, D. E. Olins, A. L. Olins, H. Herrmann, D. A. Weitz, J. Lammerding, Nuclear envelope composition determines the ability of neutrophil-type cells to passage through micron-scale constrictions. *J. Biol. Chem.* **288**, 8610–8618 (2013).

10. P. M. Davidson, C. Denais, M. C. Bakshi, J. Lammerding, Nuclear deformability constitutes a rate-limiting step during cell migration in 3-D environments. *Cell. Mol. Bioeng.* **7**, 293–306 (2014).

11. D. B. Brückner, A. Fink, C. Schreiber, P. J. F. Röttgermann, J. O. Rädler, C. P. Broedersz, Stochastic nonlinear dynamics of confined cell migration in two-state systems. *Nat. Phys.* **15**, 595–601 (2019).

12. A. Pathak, S. Kumar, Independent regulation of tumor cell migration by matrix stiffness and confinement. *Proc. Natl. Acad. Sci. U.S.A.* **109**, 10334–10339 (2012).

13. C. Beadle, M. C. Assanah, P. Monzo, R. Vallee, S. S. Rosenfeld, P. Canoll, The role of myosin II in glioma invasion of the brain. *Mol. Biol. Cell* **19**, 3357–3368 (2008).

14. Y. Fu, L. K. Chin, T. Bourouina, A. Q. Liu, A. M. J. VanDongen, Nuclear deformation during breast cancer cell transmigration. *Lab. Chip* **12**, 3774–3778 (2012).

15. A. L. McGregor, C.-R. Hsia, J. Lammerding, Squish and squeeze—The nucleus as a physical barrier during migration in confined environments. *Curr. Opin. Cell Biol.* **40**, 32–40 (2016).

16. S. Tollis, A. Rizzotto, N. T. Pham, S. Koivukoski, A. Sivakumar, S. Shave, J. Wildenhain, N. Zuleger, J. T. Keys, J. Culley, Y. Zheng, J. Lammerding, N. O. Carragher, V. G. Brunton, L. Latonen, M. Auer, M. Tyers, E. C. Schirmer, Chemical interrogation of nuclear size identifies compounds with cancer cell line-specific effects on migration and invasion. *ACS Chem. Biol.* **17**, 680–700 (2022).

17. T. Harada, J. Swift, J. Irianto, J.-W. Shin, K. R. Spinler, A. Athirasala, R. Diegmiller, P. C. D. P. Dingal, I. L. Ivanovska, D. E. Discher, Nuclear lamin stiffness is a barrier to 3D migration, but softness can limit survival. *J. Cell Biol.* **204**, 669–682 (2014).

18. M. Krause, F. W. Yang, M. te Lindert, P. Isermann, J. Schepens, R. J. A. Maas, C. Venkataraman, J. Lammerding, A. Madzvamuse, W. Hendriks, J. te Riet, K. Wolf, Cell migration through three-dimensional confining pores: Speed accelerations by deformation and recoil of the nucleus. *Philos. Trans. R. Soc. B Biol. Sci.* **374**, 20180225 (2019).

19. A. J. Lomakin, C. J. Cattin, D. Cuvelier, Z. Alraies, M. Molina, G. P. F. Nader, N. Srivastava, P. J. Sáez, J. M. Garcia-Arcos, I. Y. Zhitnyak, A. Bhargava, M. K. Driscoll, E. S. Welf, R. Fiolka, R. J. Petrie, N. S. D. Silva, J. M. González-Granado, N. Manel, A. M. Lennon-Duménil, D. J. Müller, M.

Piel, The nucleus acts as a ruler tailoring cell responses to spatial constraints. *Science* **370**, eaba2894 (2020).

20. V. Venturini, F. Pezzano, F. C. Castro, H.-M. Häkkinen, S. Jiménez-Delgado, M. Colomer-Rosell, M. Marro, Q. Tolosa-Ramon, S. Paz-López, M. A. Valverde, J. Weghuber, P. Loza-Alvarez, M. Krieg, S. Wieser, V. Ruprecht, The nucleus measures shape changes for cellular proprioception to control dynamic cell behavior. *Science* **370**, eaba2644 (2020).

21. K. Wolf, M. te Lindert, M. Krause, S. Alexander, J. te Riet, A. L. Willis, R. M. Hoffman, C. G. Figdor, S. J. Weiss, P. Friedl, Physical limits of cell migration: Control by ECM space and nuclear deformation and tuning by proteolysis and traction force. *J. Cell Biol.* **201**, 1069–1084 (2013).

22. K. Siemsen, S. Rajput, F. Rasch, F. Taheri, R. Adelung, J. Lammerding, C. Selhuber-Unkel, Tunable 3D hydrogel microchannel networks to study confined mammalian cell migration. *Adv. Healthc. Mater.* **10**, 2100625 (2021).

23. Ö. Ertekin, M. Monavari, R. Krüger, M. Fuentes-Chandía, B. Parma, G. Letort, P. Tripal, A. R. Boccaccini, A. K. Bosserhoff, P. Ceppi, M. Kappelmann-Fenzl, A. Leal-Egaña, 3D hydrogel-based microcapsules as an in vitro model to study tumorigenicity, cell migration and drug resistance. *Acta Biomater.* **142**, 208–220 (2022).

24. J. Irianto, Y. Xia, C. R. Pfeifer, A. Athirasala, J. Ji, C. Alvey, M. Tewari, R. R. Bennett, S. M. Harding, A. J. Liu, R. A. Greenberg, D. E. Discher, DNA damage follows repair factor depletion and portends genome variation in cancer cells after pore migration. *Curr. Biol.* **27**, 210–223 (2017).

25. B. S. Wong, S. R. Shah, C. L. Yankaskas, V. K. Bajpai, P.-H. Wu, D. Chin, B. Ifemembi, K. ReFaey, P. Schiapparelli, X. Zheng, S. S. Martin, C.-M. Fan, A. Quiñones-Hinojosa, K. Konstantopoulos, A microfluidic cell-migration assay for the prediction of progression-free survival and recurrence time of patients with glioblastoma. *Nat. Biomed. Eng.* **5**, 26–40 (2021).

26. D. Irimia, G. Charras, N. Agrawal, T. Mitchison, M. Toner, Polar stimulation and constrained cell migration in microfluidic channels. *Lab Chip* **7**, 1783–1790 (2007).

27. E. M. Balzer, Z. Tong, C. D. Paul, W.-C. Hung, K. M. Stroka, A. E. Boggs, S. S. Martin, K. Konstantopoulos, Physical confinement alters tumor cell adhesion and migration phenotypes. *FASEB J.* **26**, 4045–4056 (2012).

28. P. M. Davidson, J. Sliz, P. Isermann, C. Denais, J. Lammerding, Design of a microfluidic device to quantify dynamic intra-nuclear deformation during cell migration through confining environments. *Integr. Biol.* **7**, 1534–1546 (2015).

29. P. M. Davidson, A. Battistella, T. Déjardin, T. Betz, J. Plastino, N. Borghi, B. Cadot, C. Sykes, Nesprin-2 accumulates at the front of the nucleus during confined cell migration. *EMBO Rep.* **21,** e49910 (2020).

30. X. Cao, E. Moeendarbary, P. Isermann, P. M. Davidson, X. Wang, M. B. Chen, A. K. Burkart, J. Lammerding, R. D. Kamm, V. B. Shenoy, A chemomechanical model for nuclear morphology and stresses during cell transendothelial migration. *Biophys. J.* **111**, 1541–1552 (2016).

31. D. G. Thomas, A. Yenepalli, C. M. Denais, A. Rape, J. R. Beach, Y. Wang, W. P. Schiemann, H. Baskaran, J. Lammerding, T. T. Egelhoff, Non-muscle myosin IIB is critical for nuclear translocation during 3D invasion. *J. Cell Biol.* **210**, 583–594 (2015).

32. J. Keys, B. C. H. Cheung, M. A. Elpers, M. Wu, J. Lammerding, Rear cortex contraction aids in nuclear transit during confined migration by increasing pressure in the cell posterior. *J. Cell Sci.* **137**, jcs260623 (2024).

33. R. J. Ju, A. D. Falconer, K. M. Dean, R. P. Fiolka, D. P. Sester, M. Nobis, P. Timpson, A. J. Lomakin, G. Danuser, M. D. White, D. B. Oelz, N. K. Haass, S. J. Stehbens, Compression-dependent microtubule reinforcement comprises a mechanostat which enables cells to navigate confined environments. bioRxiv 2022.02.08.479516 [**Preprint**]. 29 March 2023. https://doi.org/10.1101/2022.02.08.479516.

34. S. Schmidt, P. Friedl, Interstitial cell migration: Integrin-dependent and alternative adhesion mechanisms. *Cell Tissue Res.* **339**, 83–92 (2010).

35. D. B. Brückner, M. Schmitt, A. Fink, G. Ladurner, J. Flommersfeld, N. Arlt, E. Hannezo, J. O. Rädler, C. P. Broedersz, Geometry adaptation of protrusion and polarity dynamics in confined cell migration. *Phys. Rev. X* **12**, 031041 (2022).

36. A. Afthinos, K. Bera, J. Chen, A. Ozcelikkale, A. Amitrano, M. I. Choudhury, R. Huang, P. Pachidis, P. Mistriotis, Y. Chen, K. Konstantopoulos, Migration and 3D traction force measurements inside compliant microchannels. *Nano Lett.* **22**, 7318–7327 (2022).

37. D. Selmeczi, S. Mosler, P. H. Hagedorn, N. B. Larsen, H. Flyvbjerg, Cell motility as persistent random motion: Theories from experiments. *Biophys. J.* **89**, 912–931 (2005).

38. D. B. Brückner, P. Ronceray, C. P. Broedersz, Inferring the dynamics of underdamped stochastic systems. *Phys. Rev. Lett.* **125**, 058103 (2020).

39. D. B. Brückner, C. P. Broedersz, Learning dynamical models of single and collective cell migration: A review. *Rep. Prog. Phys.* **87**, 056601 (2024).

40. I. D. Estabrook, H. R. Thiam, M. Piel, R. J. Hawkins, Calculation of the force field required for nucleus deformation during cell migration through constrictions. *PLoS Comput. Biol.* **17**, e1008592 (2021).

41. J. Flommersfeld, S. Stöberl, O. Shah, J. O. Rädler, C. P. Broedersz, Geometry-sensitive protrusion growth directs confined cell migration. *Phys. Rev. Lett.* **132**, 098401 (2024).

42. H.-R. Thiam, P. Vargas, N. Carpi, C. L. Crespo, M. Raab, E. Terriac, M. C. King, J. Jacobelli, A. S. Alberts, T. Stradal, A.-M. Lennon-Dumenil, M. Piel, Perinuclear Arp2/3-driven actin polymerization enables nuclear deformation to facilitate cell migration through complex environments. *Nat. Commun.* **7,** 10997 (2016).

43. C. Rianna, M. Radmacher, S. Kumar, Direct evidence that tumor cells soften when navigating confined spaces. *Mol. Biol. Cell* **31**, 1726–1734 (2020).

44. Z. D. Jastrzebski, *Nature and Properties of Engineering Materials* (Wiley, 1959).

45. M. Nikkhah, J. S. Strobl, E. M. Schmelz, M. Agah, Evaluation of the influence of growth medium composition on cell elasticity. *J. Biomech.* **44**, 762–766 (2011).

46. Y. Nematbakhsh, K. T. Pang, C. T. Lim, Correlating the viscoelasticity of breast cancer cells with their malignancy. *Converg. Sci. Phys. Oncol.* **3**, 034003 (2017).

47. H. Hertz, Ueber die Berührung fester elastischer Körper. *J. fur Reine Angew. Math.* **1882**, 156–171 (1882).

48. M. J. Puttock, E. G. Thwaite, *Elastic Compression of Spheres and Cylinders at Point and Line Contact* (National Standards Laboratory technical paper, CSIRO, 1969).

49. L. D. Landau, E. M. Lifshitz, *Theory of Elasticity* (Elsevier, 1986), **vol. 7**.

50. Hencky H., Über die Form des Elastizitätsgesetzes bei ideal elastischen Stoffen. *Zeit. Tech. Phys.* **9**, 215–220 (1928).

51. D. Needleman, M. Shelley, The stormy fluid dynamics of the living cell. *Phys. Today* **72**, 32–38 (2019).

52. P. Virtanen, R. Gommers, T. E. Oliphant, M. Haberland, T. Reddy, D. Cournapeau, E. Burovski, P. Peterson, W. Weckesser, J. Bright, Stéfan J. van der Walt, M. Brett, J. Wilson, K. Jarrod Millman, N. Mayorov, A. R. J. Nelson, E. Jones, R. Kern, E. Larson, C J Carey, İ. Polat, Y. Feng, E. W. Moore, J. V. Plas; SciPy 1.0 Contributors, SciPy 1.0: Fundamental algorithms for scientific computing in Python. *Nat. Methods* **17**, 261–272 (2020).

53. C. R. Harris, K. J. Millman, S. J. Van Der Walt, R. Gommers, P. Virtanen, D. Cournapeau, E. Wieser, J. Taylor, S. Berg, N. J. Smith, R. Kern, M. Picus, S. Hoyer, M. H. Van Kerkwijk, M. Brett, A. Haldane, J. F. Del Río, M. Wiebe, P. Peterson, P. Gérard-Marchant, K. Sheppard, T. Reddy, W. Weckesser, H. Abbasi, C. Gohlke, T. E. Oliphant, Array programming with NumPy. *Nature* **585**, 357–362 (2020).

54. E. Hellinger, Neue Begründung der Theorie quadratischer Formen von unendlichvielen Veränderlichen. *J. Für Reine Angew. Math.* **1909**, 210–271 (1909).

55. I.-D. Chang, On the wall effect correction of the Stokes drag formula for axially symmetric bodies moving inside a cylindrical tube. *Z. Angew. Math. Phys.* **12**, 6–14 (1961).

56. R. Phillips, J. Kondev, J. Theriot, H. G. Garcia, N. Orme, "Two-state systems: From ion channels to cooperative binding" in *Physical Biology of the Cell*, S. Scholl, Ed. (Garland Science, ed. 2, 2012), pp. 281-310.
